# Modulation of the gut microbiota and lipidomic profiles by black chokeberry (*Aronia melanocarpa* L.) polyphenols *via* the glycerophospholipid metabolism signaling pathway

**DOI:** 10.3389/fnut.2022.913729

**Published:** 2022-08-04

**Authors:** Yue Zhu, Yu-long Wei, Ioanna Karras, Peng-ju Cai, Yu-hang Xiao, Cheng-li Jia, Xiao-lin Qian, Shi-yu Zhu, Lu-jie Zheng, Xin Hu, Ai-dong Sun

**Affiliations:** ^1^College of Biological Sciences and Technology, Beijing Forestry University, Beijing, China; ^2^Beijing Key Laboratory of Forest Food Processing and Safety, Beijing Forestry University, Beijing, China; ^3^College of Agricultural, Consumer and Environmental Sciences, University of Illinois at Urbana-Champaign, Urbana, IL, United States

**Keywords:** obesity, high-fat diet, black chokeberry polyphenols extract, gut microbiota, glycerophospholipid metabolism

## Abstract

Black chokeberry (*Aronia melanocarpa* L.) is rich in polyphenols with various physiological and pharmacological activities. However, the relationship between the modulation effect of black chokeberry polyphenols on obesity and the alteration of lipid metabolism is not clearly understood. This study aimed to investigate the beneficial effects of the black chokeberry polyphenols (BCPs) treatment on the structure of gut microbiota, lipid metabolism, and associated mechanisms in high-fat diet (HFD)-induced obese rats. Here, we found that a high-fat diet promoted body weight gain and lipid accumulation in rats, while oral BCPs supplementation reduced body weight, liver, and white adipose tissue weight and alleviated dyslipidemia and hepatic steatosis in HFD-induced obese rats. In addition, BCPs supplementation prevented gut microbiota dysbiosis by increasing the relative abundance of *Bacteroides, Prevotella, Romboutsia*, and *Akkermansia* and decreasing the relative abundance of *Desulfovibrio* and *Clostridium*. Furthermore, 64 lipids were identified as potential lipid biomarkers through lipidomics analysis after BCPs supplementation, especially PE (16:0/22:6), PE (18:0/22:6), PC (20:3/19:0), LysoPE (24:0), LysoPE (24:1), and LysoPC (20:0). Moreover, our studies provided new evidence that composition of gut microbiota was closely related to the alteration of lipid profiles after BCPs supplementation. Additionally, BCPs treatment could ameliorate the disorder of lipid metabolism by regulating the mRNA and protein expression of genes related to the glycerophospholipid metabolism signaling pathway in HFD-induced obese rats. The mRNA and protein expression of PPARα, CPT1α, EPT1, and LCAT were significantly altered after BCPs treatment. In conclusion, the results of this study indicated that BCPs treatment alleviated HFD-induced obesity by modulating the composition and function of gut microbiota and improving the lipid metabolism disorder *via* the glycerophospholipid metabolism signaling pathway.

## Graphical Abstract

BCPs supplementation significantly ameliorated obesity and lipid metabolic dysfunctions through regulating gut microbial composition and glycerophospholipid metabolism signaling pathway in HFD-induced rats. 
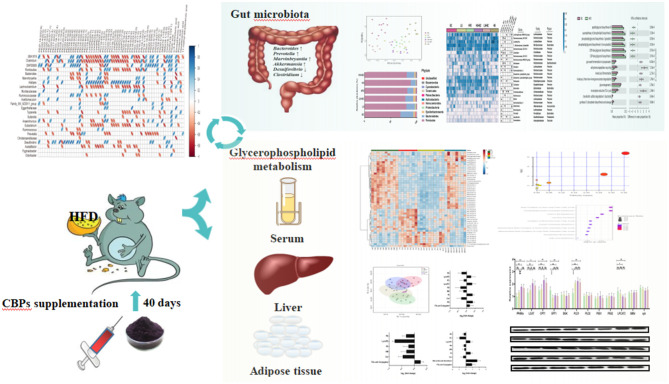

## Introduction

Over the past 15 years, the gut microbiome has been considered an important regulator of host metabolism *via* the modulation of metabolites, including the bile acids, trimethylamine N-oxide, and short-chain fatty acids by mediating the interaction between the gastrointestinal system and other organs (1, 2). Changes in the composition of gut microbiota are associated with many diseases. Obesity, dyslipidemia, type 2 diabetes mellitus (T2DM), and other metabolic diseases are related to dysbiosis of gut microbiota profiles (2). Evidence suggest that obesity and associated metabolic disorders resulted in reduced intestinal microbial richness and diversity and increased the abundance of *Firmicutes* or the ratio of *Firmicutes* to *Bacteroidetes* (F/B ratio) in high-fat diet (HFD)-fed rats (3, 4). Diet is an important external factor affecting the gut microbiota, which is considered an important option for the management of the severity of obesity and related chronic diseases (5). Black chokeberry (*Aronia melanocarpa* L.), known as “superberries,” belongs to the Rosaceae family, and originates from the eastern parts of North America and East Canada (6). Black chokeberry has been involved in numerous clinical and animal studies due to its antioxidant properties, which are in turn correlated to the high polyphenol content such as procyanidins, anthocyanins, and phenolic acids (4). In addition, polyphenols components from black chokeberry play a protective role against metabolic disorders, diabetes, and cardiovascular diseases because of supportive impacts on lipid metabolism, fasting plasma glucose, and blood pressure levels (7, 8). Previous studies have revealed that black chokeberry polyphenols supplementation could decrease the total cholesterol (TC) and triglyceride (TG) levels by altering the related hepatic gene expression in HFD-induced mice (9, 10). Moreover, the fermented black chokeberry supplementation reduced body weight and weight gain in HFD-induced mice (11). However, the mechanism of BCPs in anti-obesity and alleviating lipid metabolism disorder has not been fully elucidated.

As an important subset of metabolomics, lipidomics can be defined as the systematic characterization of lipids and their interactions in the host. Previous studies have demonstrated that lipidomics is a powerful tool to investigate lipid profile changes in some tissues (12, 13). It has been suggested that the profiling of lipidomics is closer to the disease's phenotype than that of genetic, transcriptomic, and proteomic, which may help to identify the key disease-causing regulators and biomarkers for many diseases (14). Therefore, this study aims to investigate the anti-obesity effect of the black chokeberry polyphenols (BCPs) and evaluate the role of the gut microbiota, as well as to perform a comprehensive screen of serum lipidomics, and find potential therapeutic targets and biomarkers in the HFD-induced obese rats.

## Materials and methods

### Extraction of polyphenols from black chokeberry

The BCPs were extracted in accordance with our previous research (15). Supplementary Table 1 showed the polyphenols profile of the black chokeberry. Briefly, 10 kg frozen black chokeberry were crushed using a beater for 3 min. Then, 13:7 (v/v) ethanol/water solution was added to extract polyphenols. The extraction condition was 45°C for 90 min (simultaneous with 30 min ultrasonic extraction). The solution was centrifuged at 4,000 rpm for 20 min. The supernatant was collected, and ethanol was removed from the supernatant through rotatory evaporation under vacuum at 40°C. The BCPs were then freeze-dried and stored at −80°C.

### Animals and experimental design

Wistar male rats, 6 weeks old (weighing 220 ± 20 g), were purchased from the Beijing Vital River Laboratory Animal Technology Co., Ltd. Rats were housed in specific pathogen-free (SPF) conditions and maintained in a temperature-controlled room (24°C, 60% humidity) under a 12 h-light/12 h-dark cycle. All rats were adaptively raised 1 week and randomly divided into two groups: (1) ND group (*n* = 10), fed with a control diet (10% kcal from fat, 20% kcal from proteins, 70% kcal from carbohydrates), (2) high-fat diet group, fed with a high-fat diet (45% kcal from fat, 20% kcal from proteins, 35% kcal from carbohydrates). After 2 months of continuous feeding, the obese rats model was successfully established. The high-fat diet group rats were randomly divided into five groups: (1) HFD group (*n* = 8), continually fed with HFD and administered intragastrically normal saline with 2 ml/kg body weight once daily. (2) BC group (*n* = 10), continually fed with HFD and administered intragastrically BCPs with 1,000 mg/kg body weight once daily. (3) LS group (*n* = 10), continually fed with HFD and administered intragastrically simvastatin with 5 mg/kg body weight once daily. (4) HS group (*n* = 8), continually fed with HFD and administered intragastrically simvastatin with 20 mg/kg body weight 1 time daily. (5) LS+BC group (*n* = 8), continually fed with HFD and administered intragastrically simvastatin with 5 mg/kg and BCPs with 1,000 mg/kg body weight once daily, respectively. (6) HS+BC group (*n* = 8), continually fed with HFD and administered intragastrically simvastatin with 20 mg/kg and BCPs with 1,000 mg/kg body weight 1 time daily, respectively. Animals had free access to food and water at all times. All of these treatments lasted for 6 weeks and the body weight of rats was measured weekly. At the end of the experimental period, fecal samples were collected and placed in liquid nitrogen and stored at −80°C immediately. Animals were sacrificed with carbon dioxide and blood samples were collected *via* posterior ophthalmic venous plexus. Serum was separated and stored at −80°C for biochemical analysis. The liver, kidney, spleen, heart, lung, pancreas, testicle, epididymal adipose tissue (eWAT), inguinal adipose tissue (iWAT), perirenal adipose tissue (pWAT), and mesentery adipose tissue (mWAT) were immediately removed and weighed using a precision balance. All samples were stored at −80°C for further assays.

### Blood and liver biochemical analyses

The serum was melted at 4°C for 1 h. Serum TC, TG, high-density lipoproteincholesterol (HDL-C), low-density lipoproteincholesterol (LDL-C), leptin, alanine aminotransferase (ALT), aspartate aminotransferase (AST), hepatic TC and TG were analyzed using the commercially available kits from Nanjing Jiancheng Bioengineering Institute (Nanjing, China) according to the manufacturer's instructions.

### Histopathological examinations

The method of histopathological examinations followed that of the previous study (16, 17). At room temperature, liver, eWAT, and iWAT were fixed in 4% paraformaldehyde for 24 h and then dehydrated with a sequence of ethanol solutions and embedded in paraffin. Tissue sections (5–6 μm thick) were cut and stained with hematoxylin and eosin (H&E) staining. Hepatocyte and adipocyte sizes were examined under 400× magnification and 200× magnification by a Nikon Eclipse E100 microscope (Nikon, Japan).

### Bacterial DNA extraction and 16S RRNA sequencing

Total genome DNA from 300 mg fecal samples was extracted using Magen Hipure Soil DNA Kit following the manufacturer's instructions. NGS library preparations and Illumina MiSeq sequencing were conducted by GENEWIZ, Inc. (Suzhou, China). About 20 ng DNA was used to generate amplification and V3 and V4 hypervariable regions of prokaryotic 16S rDNA were amplified. At the same time, indexed adapters were added to the ends of the 16S rDNA amplicons to generate indexed libraries ready for downstream NGS sequencing on Illumina Miseq. The PCR reactions condition are as follow: 2.5 μl of TransStart Buffer, 2 μl of dNTPs, 1 μl of each primer, and 20 ng of template DNA. Qubit 3.0 Fluorometer was used to confirm the DNA libraries concentration, which quantified the library to 10 nM. DNA libraries were multiplexed and loaded on an Illumina MiSeq instrument according to the manufacturer's instructions (Illumina, San Diego, CA, USA). Sequencing was performed using PE250/300 paired-end, image analysis and base calling were conducted by the MiSeq Control Software (MCS) embedded in the MiSeq instrument.

### Lipidomics profiling analysis

#### Sample preparation

Serum samples (200 μl each) were precipitated by adding 3 volumes of IPA pre-cooled to −20°C and mixed for 1 min. Then, the samples were incubated at room temperature for 10 min and stored overnight at −20°C to improve protein precipitation. After that, the samples were centrifuged at 12,000 g for 20 min. The supernatant was collected and analyzed by HPLC-MS.

#### Untargeted analysis of serum samples using HPLC-MS

The sample analysis was performed on an ExionLC (SCIEX, USA) coupled with a TripleTOF 5,600+(SCIEX, USA) in both positive and negative ionization modes. The column was a 2.1 × 100 mm, 2.6 μm, C18 100Å column (Phenomenex, CHN), and the temperature was maintained at 45°C. Mobile phase A consisted of H_2_O/ACN/MeOH (3:1:1, v:v:v) mixed with 5 mM ammonium acetate and mobile phase B consisted of IPA mixed with 5 mM ammonium acetate. The flow rate was 0.3 ml/min. The injection volume was 2 μl. The separation was conducted under the following gradient: 0 min 30% B; 0–1 min 30% B; 1–14 min 95% B; 14–17 min 95% B; 17–17.1 min 30% B; 17.1–20 min 30% B. The QTOFMS instrument was operated in electrospray ionization by using IDA high-sensitivity scanning mode with the following parameters: curtain gas, 35 psi; ion source gas 1, 60 psi; ion source gas 2, 60 psi; temperature, 600 °C for the positive mode and 550°C for the negative mode; ion spray voltage floating, 5.5 KV for the positive mode and 4.5 KV for the negative node. The scanning time was 20 min; scan range was from 100 to 1,200 m/z for MS scan and 50–1,200 m/z for MS/MS scans. Twelve ions from the MS scan were selected for MS/MS scan. The MS/MS accumulation time was 0.05 s, the collision energy was 40, and the collision energy range is the theoretical frequency ± 20.

### Real-time PCR analysis

An RNeasy kit (SinoGene Biotech co., Ltd. China) was used to extract total RNA from the liver and eWAT. Thermo First cDNA Synthesis Kit (SinoGene Biotech co., Ltd. China) was used for reverse transcription. Gene expression was relatively quantified with SYBR green probe using a StepOnePLUS Real-Time PCR System (Thermo Fisher Scientific Inc. Waltham, MA USA). β-actin gene was applied as reference. Primer sequences were listed in Supplementary Table 2.

### Western blot analysis

RIPA lysis buffer (P0013B, Beyotime, China) was used to extract the protein in liver tissues. Protein was separated on SDS-PAGE and then transferred to polyvinylidene fluoride (PVDF) membranes. The PVDF membranes were immersed in TBS-Tween containing 5% skimmed milk powder at room temperature on a shaker for 2 h and incubated overnight with primary antibodies at 4°C. The membrane was washed four times and incubated with secondary antibodies for 1 h. The membrane was washed 4 times and the protein bands were visualized using Western Chemiluminescent HRP Substrate (Millipore, USA).

### Statistics analysis

Statistical analysis was performed by using SPSS 21 software and GraphPad Prism version 8.4. One-way ANOVA was employed to calculate the differences between multiple groups with Tukey's *post-hoc* test. *p* < 0.05 were considered statistically significant. All data are expressed as the mean ± SEM.

The QIIME data analysis package was used for 16S rRNA data analysis. The forward and reverse reads were joined and assigned to samples based on barcode and then truncated by cutting off the barcode and primer sequence. Quality filtering on joined sequences was performed and sequences that did not fulfill the following criteria were discarded: sequence length > 200 bp, no ambiguous bases, mean quality score ≥ 20. Then the sequences were compared with the reference database (RDP Gold database) using the UCHIME algorithm to detect chimeric sequences, which were removed. Next, sequences were grouped into operational taxonomic units (OTUs) based on VSEARCH (1.9.6) against the Silva 132 database at a similarity of 97%. The Ribosomal Database Program (RDP) classifier was used to assign a taxonomic category to all OTUs at a confidence threshold of 0.8. α-diversity and β-diversity statistics were calculated in QIIME using the Shannon index, Simpson index, Chao1 index, and ACE. The abundance of annotated OTUs was performed to the linear discriminant analysis (LDA) effect size (LEfSe) online tool (http://huttenhower.sph.harvard.edu/galaxy/root?tool_id=lefse_upload).

The lipidomics raw data were imported to the Progenesis QI (Waters) for peak alignment to obtain a peak list containing the retention time, m/z, and peak area of each sample. Open database sources, including lipid maps, the Human Metabolome Database(HMDB), and the National Institute of Standards and Technology (NIST) were used to identify compounds.

## Results

### Effect of BCPs treatment on body weight, dyslipidemia, and liver steatosis of HFD-induced obese rats

**Figure 1:**
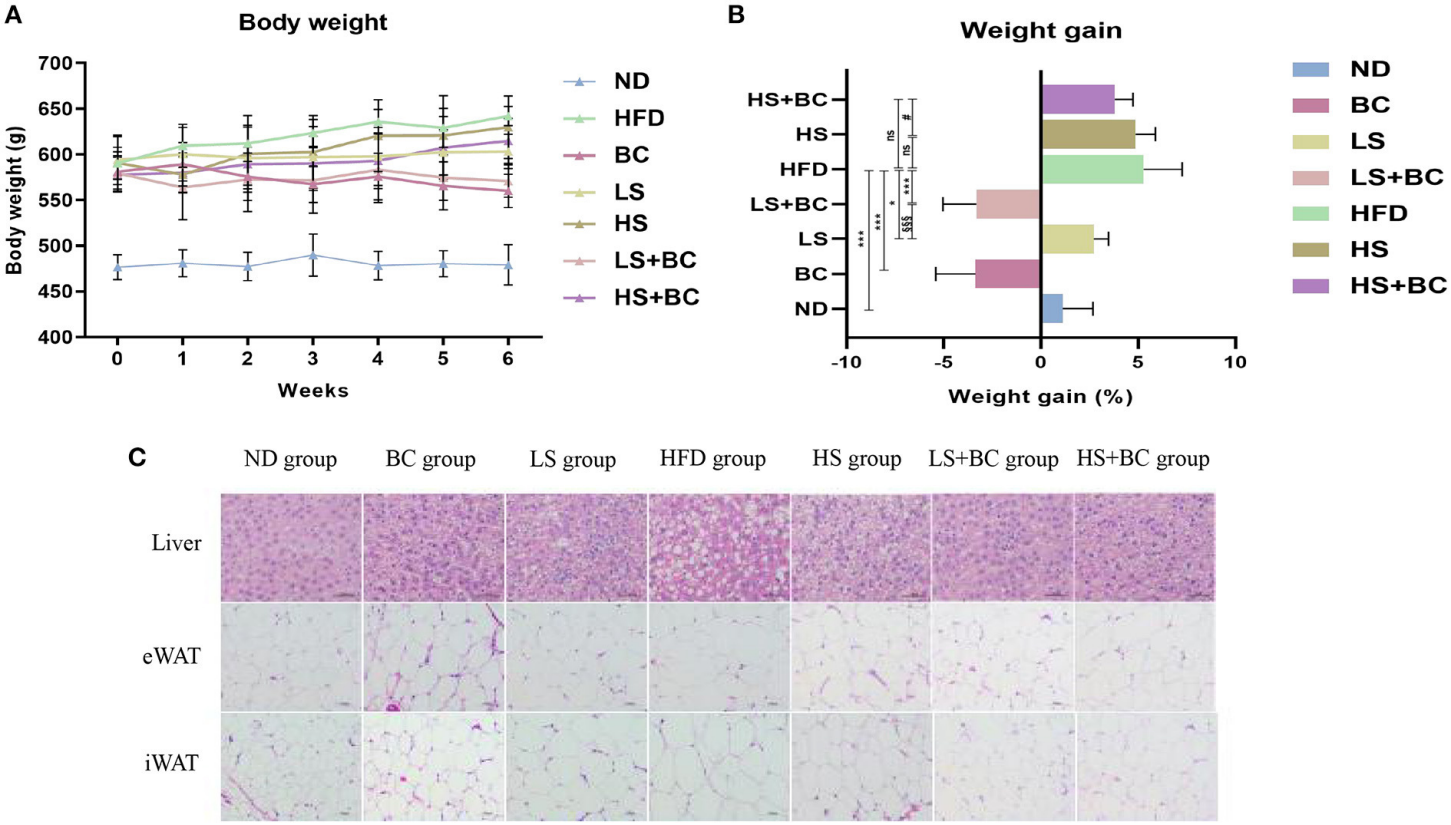
Effect of BCPs treatment on body weight and liver steatosis in HFD-induced obese rats. **(A)** body weight (g), **(B)** weight gain (%), **(C)** histological analysis for liver and adipose tissues. Values are means ± SEMs. *p < 0.05, **p < 0.01 and ***p < 0.001 when comparing other groups with HFD group, ^#^*p* < 0.05, ^##^*p* < 0.01, ^###^*p* < 0.001 when comparing HS group with HS + BC group, ^§^*p* < 0.05, ^§§^*p* < 0.01, ^§§§^*p* < 0.001 when comparing LS group with LS+BC group.

As shown in Figure 1A, the body weight decreased gradually during BCPs treatment. Compared with the HFD group, weight gain was dramatically reduced in the BC group after 6 weeks (*p* < 0.001) (Figure 1B). The trend of body weight change in the LS+BC group was similar to that in the BC group. The body weight of the HS group and HS+BC group increased slowly and weight gain was 4.87 and 3.80%, respectively, which was not significantly altered compared to the HFD group (Figures 1A,B). However, there was a significant difference in weight gain between the HS group and the HS+BC group (*p* < 0.05). Similar results were also found between the LS group and LS+BC group (*p* < 0.05). Simultaneously, as shown in Table 1, compared to the HFD group, iWAT and pWAT weights were decreased in the six groups. Moreover, only the control group, BC group, and LS+BC group had a lower eWAT and mWAT weight than that of the HFD group. Except for the HS+BC group, the liver weight was reduced in all other groups compared to the HFD group. There were no significant differences in the weight of the heart, kidney, spleen, lung, pancreas, and testicles among these groups (Supplementary Figure 1). Furthermore, we next measured TC and TG concentration and observed H&E staining results in liver and adipose tissues. The TC levels in the liver were significantly reduced in the LS group, HS group, and HS+BC group compared to the HFD group (*p* < 0.001), whereas there was no significant difference in TG levels among the four groups. TC and TG levels were decreased in both the BC group and LS+BC group compared to the HFD group (*p* = 0.01 and *p* < 0.05, respectively) (Table 1). Similarly, H&E staining of liver and white adipose tissues also showed that obese rats treated with BCPs had significantly reduced hepatic fat droplets and adipocyte size (Figure 1C) in the liver, and improving liver steatosis was better than that of low-dose simvastatin treatment alone. Interestingly, increasing the simvastatin concentration did not significantly attenuate liver steatosis and lipid accumulation in white adipose tissues, which was not indicated dose-dependent manner in obese rats.

**Table 1:** The liver weight, adipose tissues weight, liver and serum biochemical concentrations of TC, TG, HDL-C, LDL-C, leptin, AST and ALT in rats.

	**ND**	**HFD**	**BC**	**LS**	**HS**	**LS+BC**	**HS+BC**
Liver weight (g)	14.16 ± 1.73^d^	25.75 ± 3.25^a^	18.61 ± 1.52^c^	21.29 ± 3.18^b^	22.62 ± 4.34^ab^	17.78 ± 2.17^c^	19.56 ± 2.93^bc^
eWAT weight (g)	11.75 ± 2.89^c^	25.68 ± 7.48^a^	19.48 ± 3.66^b^	21.85 ± 3.01^ab^	21.15 ± 5.57^ab^	20.20 ± 2.33^b^	20.92 ± 5.41^ab^
iWAT weight (g)	8.71 ± 5.25^d^	31.97 ± 6.43^a^	14.27 ± 3.52^c^	19.34 ± 4.07^b^	18.57 ± 5.81^b^	14.71 ± 2.98^c^	16.50 ± 3.72^bc^
pWAT weight (g)	11.91 ± 5.28^d^	33.06 ± 4.27^a^	22.84 ± 3.75^c^	26.96 ± 3.32^b^	25.81 ± 5.02^bc^	20.93 ± 4.17^c^	24.26 ± 5.05^bc^
mWAT weight (g)	4.92 ± 1.33^c^	13.30 ± 4.06^a^	8.49 ± 2.62^b^	11.35 ± 3.49^ab^	10.30 ± 2.59^ab^	8.68 ± 2.01^b^	9.68 ± 4.39^b^
Liver TC (mmol/gprot)	0.084 ± 0.02^d^	0.20 ± 0.02^a^	0.16 ± 0.02^b^	0.15 ± 0.02^b^	0.13 ± 0.01^c^	0.14 ± 0.01^bc^	0.13 ± 0.02^c^
Liver TG (mmol/gprot)	0.25 ± 0.02^c^	0.33 ± 0.04^a^	0.26 ± 0.03^a^	0.31 ± 0.02^a^	0.34 ± 0.03^b^	0.28 ± 0.03^a^	0.31 ± 0.03^bc^
Serum TC (mmoL/L)	3.19 ± 0.26^c^	4.56 ± 0.56^a^	3.67 ± 0.41^b^	3.14 ± 0.57^c^	3.32 ± 0.72bc	3.35 ± 0.53^bc^	3.41 ± 0.62^bc^
Serum TG (mmoL/L)	1.07 ± 0.22^c^	1.58 ± 0.24^a^	1.18 ± 0.21^b^	1.28 ± 0.18^ab^	1.60 ± 0.64^a^	1.17 ± 0.28^bc^	1.38 ± 0.49^ab^
Serum HDL-C (mmoL/L)	3.20 ± 0.27^a^	1.81 ± 0.38^d^	2.86 ± 0.31^b^	2.16 ± 0.22^c^	2.24 ± 0.57^c^	2.61 ± 0.24^b^	2.35 ± 0.44^c^
Serum LDL-C (mmoL/L)	0.72 ± 0.08^c^	1.47 ± 0.41^a^	0.88 ± 0.20^b^	0.82 ± 0.14^bc^	0.91 ± 0.21^b^	0.68 ± 0.31^c^	0.79 ± 0.17^bc^
Serum leptin (ng/uL)	4.15 ± 0.75	4.90 ± 0.63	5.29 ± 1.02	4.99 ± 0.78	5.02 ± 0.55	4.59 ± 0.48	4.98 ± 0.51
Serum AST (mmoL/L)	426.12 ± 60.23^b^	456.00 ± 55.14^b^	470.22 ± 50.41^b^	463.14 ± 69.17^b^	568.50 ± 41.32^a^	414.25 ± 33.57^b^	396.38 ± 30.25^b^
Serum ALT (mmoL/L)	44.25 ± 8.76	62.17 ± 7.17	54.25 ± 8.95	51.00 ± 10.89	63.14 ± 6.47	62.50 ± 6.25	68.63 ± 7.25

Data are shown as the mean ± SEM.

eWAT, epididymal adipose tissue; iWAT, inguinal adipose tissue; pWAT, perirenal adipose tissue; mWAT, mesentery adipose tissue.

One-way ANOVA was conducted to compare these groups. Different letters indicate difference between groups.

After 6 weeks of HFD treatment, the serum TC, TG, and LDL-C levels were observably increased and the serum HDL-C levels were decreased in the HFD group compared to the ND group. BCPs treatment significantly attenuated dyslipidemia in obese rats by decreasing the TC, TG, and LDL-C levels and increasing HDL-C levels (Table 1). Both high-dose simvastatin and low-dose simvastatin treatment could dramatically reduce TC and LDL-C levels in obese rats. As previously reported, statin therapy is associated with significantly lower TC and LDL-C levels, which is achieved through inhibiting HMG-CoA reductase activities. However, there was no significant difference in TG and HDL-C levels among the HFD group, LS group, and HS group. Furthermore, in terms of reducing serum TG and increasing HDL-C levels, the effect of low-dose simvastatin combined with BCPs treatment was better than high-dose simvastatin combined with BCPs treatment. In addition, there was no significant difference in serum leptin and ALT levels among these groups, whereas the serum AST level was observably increased in the HS group compared to the other groups.

### Effect of BCPs treatment on the gut microbiota of HFD-induced obese rats

**Figure 2:**
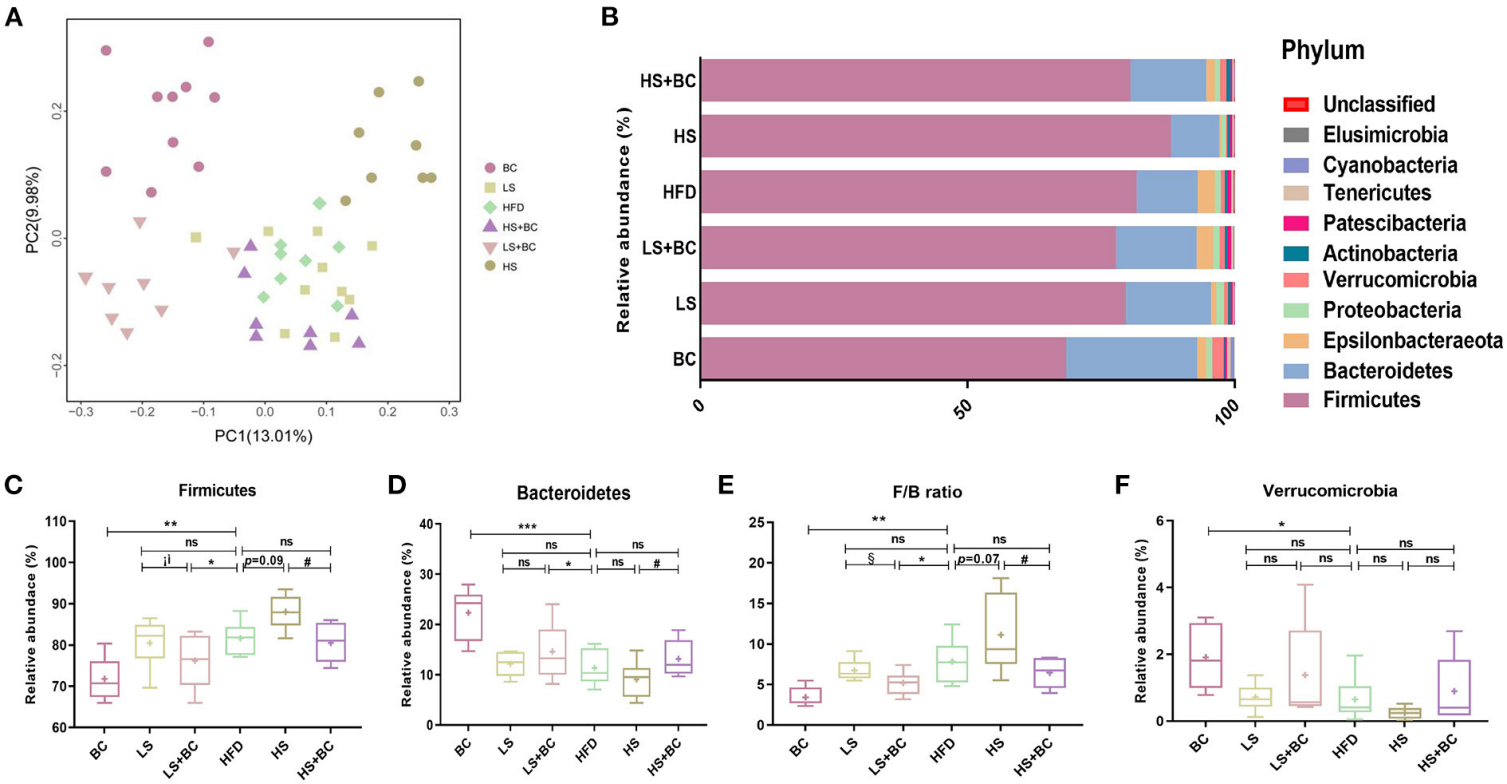
Effect of BCPs treatment on gut microbiota structure of phylum level in HFD-induced obese rats. **(A)** Principal coordinates analysis (PCoA) of gut microbiota between the six groups, **(B)** compositional change at the phylum level, **(C–F)** the relative abundance of *Firmicutes, Bacteroidetes, Verrucomicrobia* and F/B ratio in the six groups. Values are means ± SEMs. *p < 0.05, **p < 0.01 and ***p < 0.001 when comparing other groups with HFD group, ^#^*p* < 0.05, ^##^*p* < 0.01, ^###^*p* < 0.001 when comparing HS group with HS+BC group, ^§^*p* < 0.05, ^§§^*p* < 0.01, ^§§§^*p* < 0.001 when comparing LS group with LS+BC group.

16S rRNA gene sequencing was performed to measure the structure and composition of gut microbiota in HFD-induced obese rats. As shown in Supplementary Figure 2, there were no significant differences in the ACE index, Chao1 index, Shannon index, and Simpson index among the six groups (BC group, LS group, HS group, HFD group, LS+BC group, and HS+BC group). To reveal the effect of BCPs treatment on gut microbial structure, principal coordinates analysis (PCoA) based on unweighted Unifrac distance was used to evaluate the structural changes among the six groups (Figure 2A). The PCoA analysis result indicated that gut microbiota structure of the BC group, LS+BC group, and HS group was separated from the HFD group. However, there was no significant separation of gut microbiota structure among the LS group, HS+BC group, and HFD group. This result demonstrated that the BCPs treatment, high-dose simvastatin treatment alone, and low-dose simvastatin combined with BCPs treatment significantly influenced the gut microbiota structure in HFD-induced obese rats.

**Figure 3:**
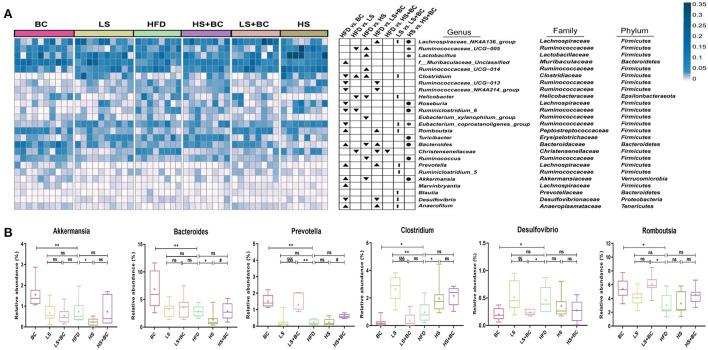
Effect of BCPs treatment on gut microbiota structure of genera level in HFD-induced obese rats. **(A)** Heatmap showing the abundance of 25 genera significantly altered after BC treatment; ▴ represent more abundant in BC group, LS group, HS group, LS+BC group and HS+BC group relative to HFD group; ▾ represent less abundant in BC group, LS group, HS group, LS+BC group and HS+BC group relative to HFD group; ↑ represent more abundant in LS+BC group relative to LS group; ↓ represent less abundant in LS+BC group relative to LS group; • represent more abundant in HS+BC group relative to HS group; 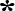 represent less abundant in HS+BC group relative to HS group; **(B)** Changes in the relative abundance of representative genera. Values are means ± SEMs. *p < 0.05, **p < 0.01 and ***p < 0.001 when comparing other groups with HFD group, ^#^*p* < 0.05, ^##^*p* < 0.01, ^###^*p* < 0.001 when comparing HS group with HS+BC group, ^§^*p* < 0.05, ^§§^*p* < 0.01, ^§§§^*p* < 0.001 when comparing LS group with LS+BC group.

At the phylum level, the relative abundance of *Bacteroidetes* increased and the relative abundance of *Firmicutes* decreased in the BC group and LS+BC group compared to the HFD group (Figures 2B–D). Moreover, the F/B ratio markedly decreased in the BC group and LS+BC group compared to the HFD group (*p* < 0.01 and *p* < 0.05, respectively) (Figure 2E). Notably, the relative abundance of *Verrucomicrobia* in the BC group was higher than that of HFD group (Figure 2F). In addition, compared with the HFD group, the relative abundance of *Bacteroidetes, Firmicutes, Verrucomicrobia*, and F/B ratio did not change in the LS group, HS group, and HS+BC group (Figures 2C–F). At the genus level, the relative abundance of *Bacteroides, Romboutsia, Prevotella, Akkermansia, Marvinbryantia*, and *Anaerofilum* was sharply increased, whereas the relative abundance of *Clostridium, Ruminococcaceae_UCG-013, Ruminococcaceae_NK4A214_group, Roseburia, Desulfovibrio, Ruminiclostridium_6*, and *Eubacterium_coprostanollgenes_group* was decreased in the BC group compared to the HFD group (Figures 3A,B). Moreover, the relative abundance of *Lachnospiraceae_NK4A136_group, Bacteroides, Prevotella, Romboutsia*, and *Anaerofilum* was increased and the relative abundance of *Ruminococcaceae_UCG-013, Ruminococcaceae_NK4A214_group, Christensenellaceae*, and *Desulfovibrio* was lowered in LS+BC group compared to the HFD group. The relative abundance of *Ruminococcaceae_UCG-005, Clostridium, Helicobacter, Ruminiclostridium_6*, and *Christensenellaceae* was significantly changed in the LS group compared to the HFD group. However, there was no significant difference in the relative abundance of genera between the HS+BC group and the HFD group. Furthermore, the relative abundance of beneficial genus *Bacteroides* and *Akkermansia* was reduced and the relative abundance of the conditional pathogenic genus *Clostridium* was increased in the HS group compared to the HFD group.

**Figure 4:**
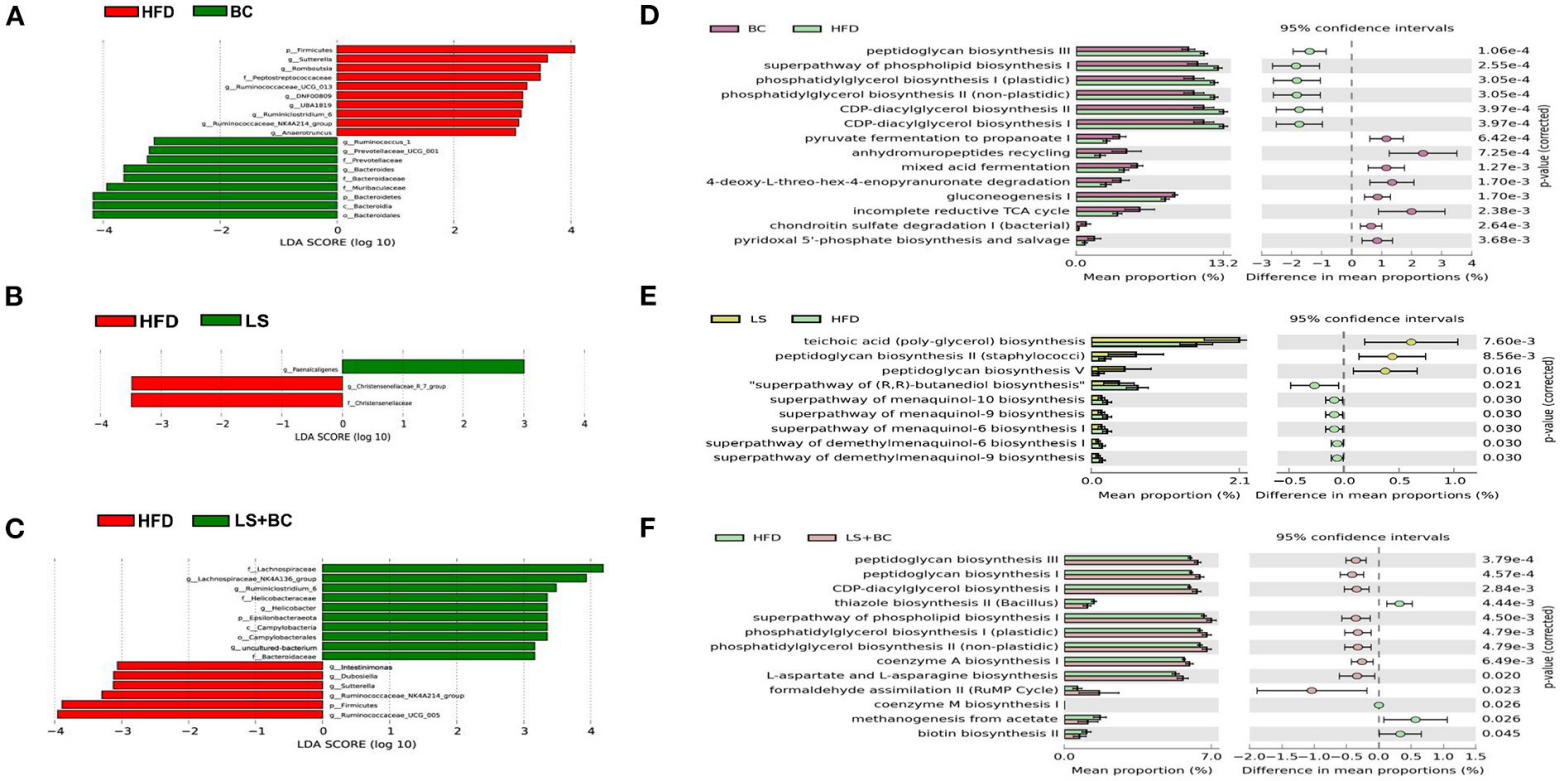
Different genera and metabolic pathways were found between BC group, LS group, and LS+BC group. **(A–C)** Linear discriminant analysis (LDA) scores derived from LEfSe analysis, showing the biomarker taxa, **(D–F)** prediction of the function of microbial genes involved in metabolism by PICRUSt analysis and based on the Welch's *t-*test (*p* < 0.05). The colored circles represent the 95% confidence intervals calculated by Welch's inverted method.

The LEfSe analysis was used to identify the biomarkers with significant differences between the two groups. As shown in Figure 4A and Supplementary Figure 3A, compared with the BC group, the relative abundance of *Sutterella, Romboutsia, Ruminococcaceae_UCG-013, UBA1819, Anaerotruncus*, and *DNF00809* was altered significantly in the HFD group. However, the relative abundance of *Bacteroides, Prevotella*, and *Ruminococcus_1* had significant differences in the BC group compared to the HFD group. Similarly, there was a significant difference in the relative abundance of *Intestinimonas, Sutterella, Dubosiella, Ruminococcaceae_UCG-005*, and *Ruminococcaceae_NK4A214_group* between the LS+BC group and the HFD group (Figure 4C and Supplementary Figure 3C). In addition, the relative abundance of *Paenalcaligenes* and *Christensenellaceae_r_7_group* were changed between the LS group and HFD group (Figure 4B). Only one genus *Turicibacter* was significantly changed in the HS+BC group compared to the HFD group (Supplementary Figure 3D). Besides, compared with HFD group, there was no significant difference in the relative abundance of genus level in the HS group (Supplementary Figure 3E). The result was consistent with the above analysis of genus level. Therefore, the results indicated that BCPs combined with high-dose simvastatin treatment, as well as high-dose simvastatin treatment alone failed to alter the composition of beneficial bacteria in the intestine and inhibit obesity. Moreover, PICRUSt analysis was used to predict the function of microbial genes involved in the metabolism pathway in the HFD group, BC group, LS group, and LS+BC group. As shown in Figures 4D–F, the results of the PICRUSt analysis suggested that the CDP-diacylglycerol biosynthesis I pathway, CDP-diacylglycerol biosynthesis II pathway, phosphatidylglycerol biosynthesis I (plastidic) pathway, phosphatidylglycerol biosynthesis II (non-plastidic) pathway and super pathway of phospholipid biosynthesis I pathway were mainly changed in the BC group compared to the HFD group (Figure 4D). The similar result was found between LS+BC group and HFD group (Figure 4F). In the LS group, teichoic acid (poly-glycerol) biosynthesis pathway and peptidoglycan biosynthesis II (staphylococci) pathway were changed compared to the HFD group (Figure 4E). Hence, we assumed that BCPs treatment may improve the disorder of lipid metabolism by reshaping the structure and function of gut microbiota, and further inhibit obesity. Next, untargeted lipidomic analysis was used to explore the effect of BCPs treatment on lipid metabolism in HFD-induced obese rats.

### Effect of BCPs treatment on lipid profiles in serum of HFD-induced obese rats

**Figure 5:**
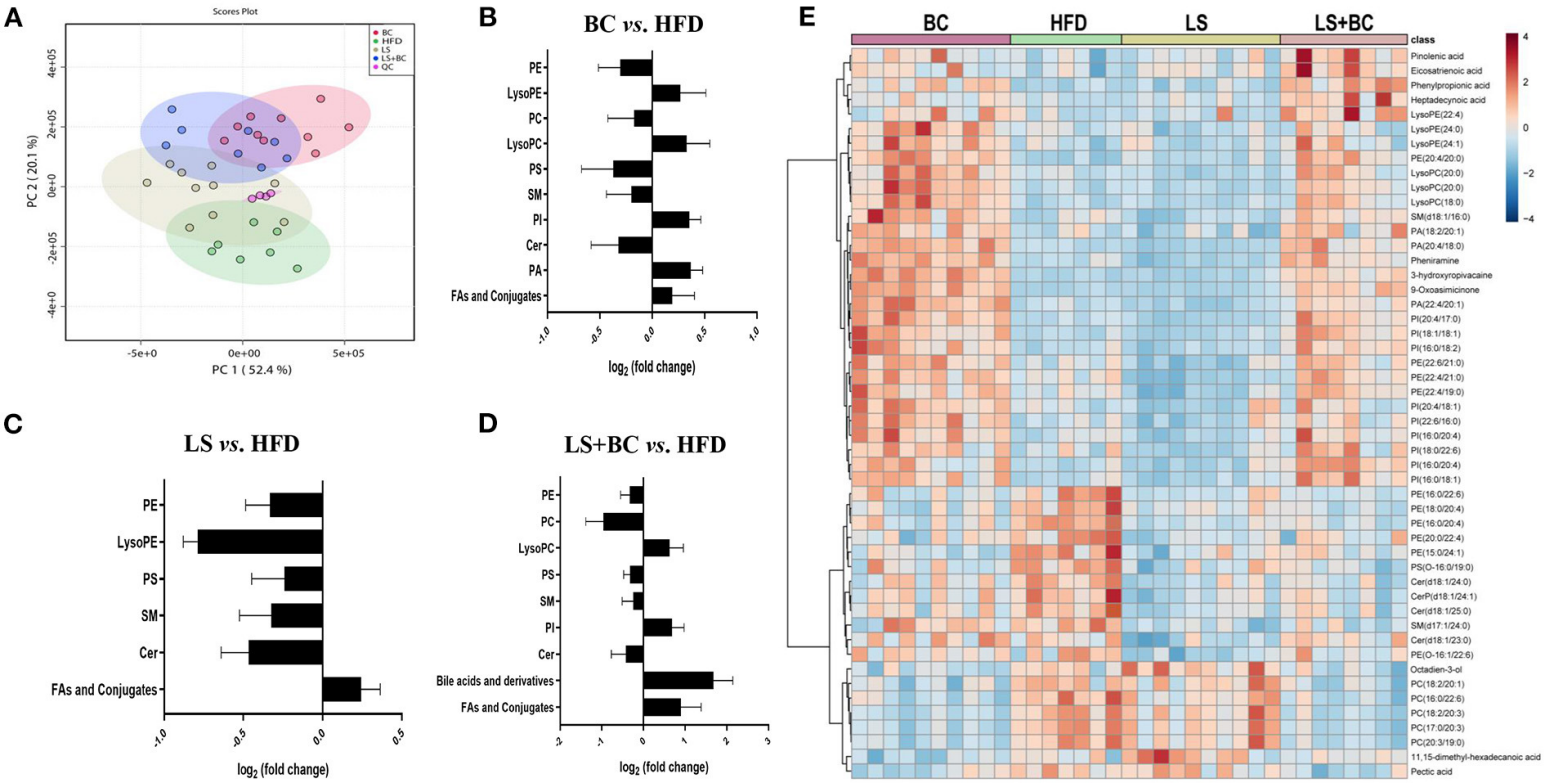
Effect of BCPs treatment on lipid profiles of serum in HFD-induced obese rats. **(A)** A two-dimensional principal component analysis (PCA) plot was generated with MetaboAnalyst 4.0 using the peak intensity data, **(B–D)** total lipids in the serum of BC group *vs*. HFD group, LS group *vs*. HFD group and LS+BC group *vs*. HFD group, respectively. The calculation of fold changes was based on the sum of all intensities of identified lipids of each class. **(E)** Heat map and hierarchical clustering of 50 identified differential lipids in the four groups.

To study the effect of BCPs treatment on lipid profiles in HFD-induced obese rats, we performed untargeted lipidomics analysis in the BC group, LS group, LS+BC group, and HFD group. Based on the OPLS-DA models of lipid profiles in serum, the levels of R^2^Y and Q^2^ were 99 and 76%, respectively (Supplementary Figure 4A). There was a clear classification between the BC group and the HFD group, demonstrating that lipid profiles were significantly distinguished between the two groups. Similarly, the LS+BC group and HFD group were obviously clustered and the levels of R^2^Y and Q^2^ were 99 and 80%, respectively (Supplementary Figure 4C). However, the level of the OPLS-DA model in the LS group and HFD group were slightly lower and the values of R^2^Y and Q^2^ were 92 and 54%, respectively (Supplementary Figure 4B). In addition, principle component analysis (PCA) was performed to analyze the lipidomics data (Figure 5A). The BC group and LS+BC group showed clear trends of separation from the HFD group.

**Figure 6:**
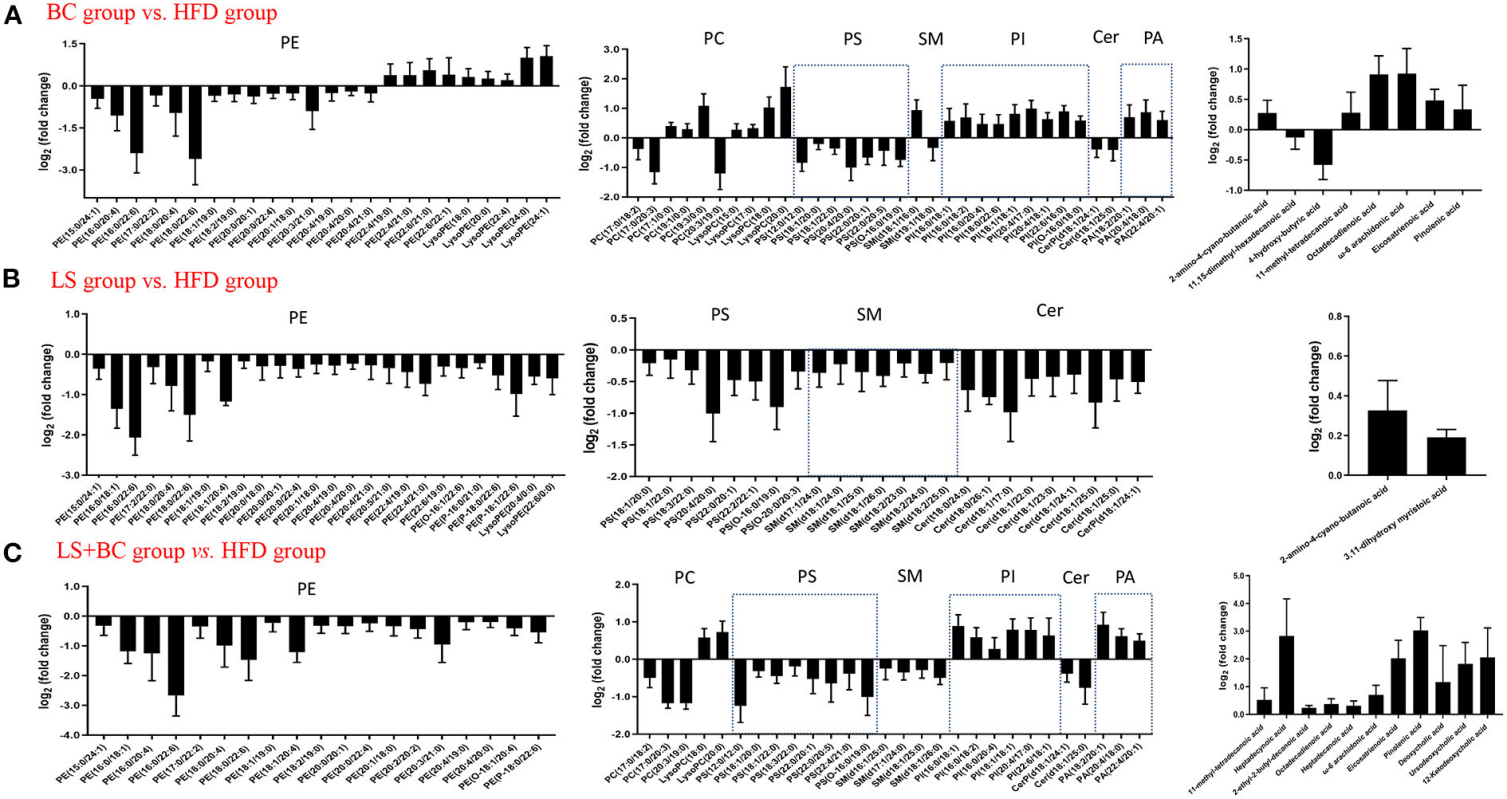
Subspecies level analysis of the potential lipid biomarkers responsible for anti-obesity and improving lipid profiling after CBPs treatment. **(A)** BC group *vs*. HFD group, **(B)** LS group *vs*. HFD group, **(C)** LS+BC group *vs*. HFD group. PE, phosphatidylethanolamines; PC, phosphatidylcholines; PS, phosphatidylserine; SM, sphingomyelin; PI, phosphatidylinositol; Cer, ceramides; PA, phosphatidic acids.

Further, we analyzed the change in metabolites between the two groups using lipidomics analysis and selected metabolites with a VIP (variable importance of projection)>1 and *p* < 0.05. Sixty-four lipid species were significantly changed in the BC group compared to the HFD group and were listed in Supplementary Table 3. Generally, the levels of phosphatidylethanolamine (PE), phosphatidylcholine (PC), phosphatidylserine (PS), sphingomyelin (SM), and Ceramides (Cer) were decreased, whereas the levels of all phosphatidylinositols (PI), phosphatidic acid (PA), lysophosphatidylethanolamine (LysoPE), lysophosphatidylcholine (LysoPC), and fatty acids (FAs) and conjugates were enhanced in the BC group compared to the HFD group (Figure 5B). Furthermore, most of the individual PE species were obviously reduced in the BC group. The levels of PE(16:0/22:6) and PE(18:0/22:6) were significantly lower, while the levels of PE(22:4/19:0), PE(22:4/21:0), PE(22:6/21:0) and PE(22:6/22:1) were increased in the BC group compared to the HFD group (Figure 6A). In addition, seven PS species [PS(12:0/12:0), PS(18:1/20:0), PS(18:0/22:0), PS(20:4/20:0), PS(22:0/20:0), PS(22:0/20:0), and PS(O-16:0/19:0)] and 2 Cer species [Cer(d18:1/24:1) and Cer(d18:1/25:0)] were decreased in the BC group compared to the HFD group. Conversely, nine PI species [PI(16:0/18:1), PI(16:0/18:2), PI(16:0/20:4), PI(18:0/22:6), PI(18:1/18:1), PI(20:4/17:0), PI(20:4/18:1), PI(22:6/16:0) and PI(O-16:0/18:0)] and three PA species [PA(18:2/20:1), PA(20:4/18:0), and PA(22:4/20:1)] were increased after BCPs treatment. Besides, an elevation in serum levels of five LysoPE species [LysoPE(18:0), LysoPE(20:0), LysoPE(22:4), LysoPE(24:0) and LysoPE(24:1)] and four LysoPC species [LysoPC(15:0), LysoPC(17:0), LysoPC(18:0), and LysoPC(20:0)] was observed in the BC group compared to the HFD group. Interestingly, some PC and SM individual species were elevated, such as PC(17:1/0:0), PC(19:1/0:0), PC(19:3/0:0) and SM(d18:1/16:0), while some PC and SM individual species [PC(17:0/18:2), PC(17:0/20:3), PC(20:3/19:0), and SM(d19:1/18:0)] were reduced in the BC group compared to the HFD group. Additionally, eight FAs and conjugates were altered in the BC group. Among them, polyunsaturated fatty acids, octadecadienoic acid, ω-6 arachidonic acid, eicosatrienoic acid, and pinolenic acid, were increased in the BC group (Figure 6A). Notably, 52 individual lipid species in the LS group were notably distinguished compared to the HFD group's species (Supplementary Table 4). Compared to the HFD group, the level of Cer, PE, PS, SM, LysoPE, LysoPC, and FAs, and conjugates were significantly reduced in the LS group (Figure 5C). Specifically, the level of PE(16:0/18:1), PE(16:0/22:6), PE(18:0/22:6), PE(18:1/20:4), and PE(P-18:1/22:6) were obviously decreased in the LS group compared to the HFD group (Fold change (FC)>2) (Figure 6B). In addition, 60 individual lipid species were significantly changed in the LS+BC group (Supplementary Table 5). Among of them, PE, PC, PS, SM and Cer species were decreased in the LS+BC group (Figure 5D). In detail, the level of five PE species [PE(16:0/18:1), PE(16:0/20:4), PE(16:0/22:6), PE(18:1/20:4), PE(18:0/22:6)], two PC species [PC(17:0/20:3) and PC(20:3/19:0)] and 2 PS species [PS(12:0/12:0) and PS(O-16:0/19:0)] were significantly reduced (FC>2), whereas the level of 2 LysoPC species [LysoPC(18:0) and LysoPC(20:0)], six PI species [PI(16:0/18:1), PI(16:0/18:2), PI(16:0/20:4), PI(18:1/18:1), PI(20:4/17:0), and PI(22:6/18:1)] and 3 PA species [PA(18:2/20:1), PA(20:4/18:0) and PA(22:4/20:1)] were increased in the LS+BC group compared to the HFD group. Moreover, eight FAs and conjugates and three bile acids and derivatives were higher in the LS+BC group compared to the HFD group. In particular, heptadecanoic acid, eicosatrienoic acid, pinolenic acid, deoxycholic acid, ursodeoxycholic acid, and 12-ketodeoxycholic acid were sharply increased in the LS+BC group compared to the HFD group (FC>2) (Figure 6C).

To show the overall variation in the lipid profiles of the four groups, a heatmap was built and based on the 50 identified endogenous metabolites. As shown in Figure 5E, an obvious separation of the identified lipids was displayed between the four groups. Four FAs and conjugates species (pinolenic acid, eicosatrienoic acid, phenylpropionic acid, and heptadecynoic acid) were increased in the BC group and the LS+BC group. Moreover, a significant number of lipids were up-regulated in the BC group and LS+BC group in comparison with the HFD group and LS group, including three LysoPE species, three LysoPC species, nine PI species, and three PA species. In particular, PE species [(PE(20:4/20:0), PE(22:6/21:0), PE(22:4/21:0), and PE(22:4/19:0)] composed of long-chain high unsaturated fatty acids at the C-1 position were significantly increased in the BC group and LS+BC group, whereas the C-2 position consisting of long-chain high unsaturated fatty acids of six PE species [PE(16:0/22:6), PE(18:0/20:4), PE(16:0/20:4), and PE(20:0/22:4)] were elevated in the HFD group. Moreover, the level of 4 ceramides species [Cer(d18:1/24:0), CerP(d18:1/24:1), Cer(d18:1/25:0), and Cer(d18:1/23:0)] were positively associated with the HFD group. Besides, five PC species and two FAs and conjugates species were also higher in the LS group and BC group, including PC(18:2/20:1), PC(16:0/22:6), PC(18:2/20:3), PC(17:0/20:3), PC(20:3/19:0), 11,15-dimethyl-hexadecanoic acid and pectic acid (Figure 5E).

**Figure 7:**
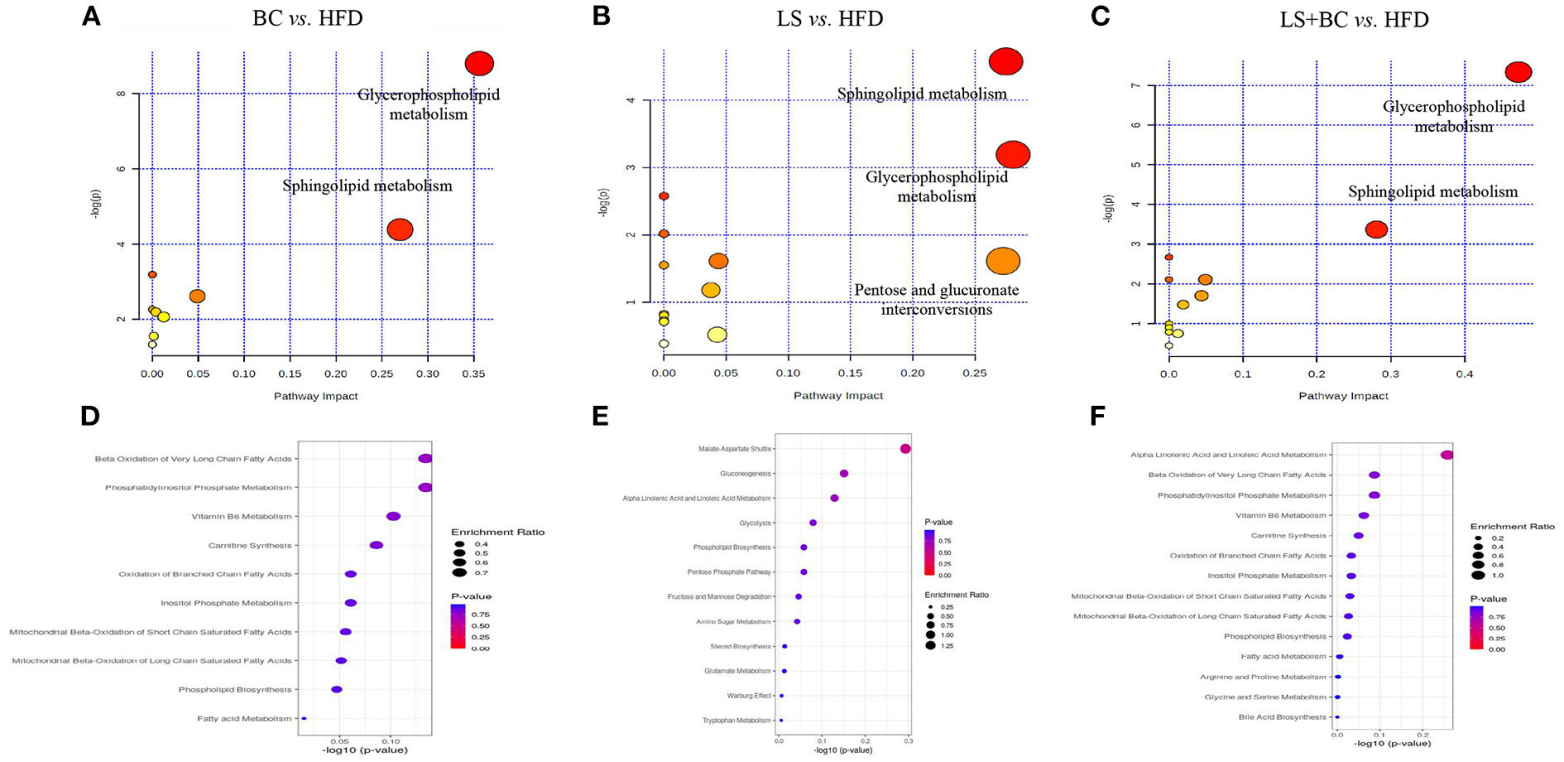
Lipid metabolic pathway analysis based on significantly differential lipid species in serum. **(A–C)** All matched pathways are plotted according to *p*-value from pathway enrichment analysis and pathway impact score from pathway topology analysis. The color and size of each circle represented the *p*-value and the pathway impact factor, respectively. **(D–F)** Over Representation Analysis (ORA) is performed and implemented using the hypergeometric test to evaluate whether a particular metabolite set is represented more than expected by chance within the lipid metabolite. One-tailed *p*-values are provided after adjusting for multiple testing. Enrichment ratio is computed by hits / expected. The color and size of each circle represented the *p*-value and the enrichment ratio, respectively.

Furthermore, MetaboAnalyst 4.0 software was used for metabolic pathways analysis and enrichment analysis. The results were shown in Table 2 and Figure 7. Two main pathways, glycerophospholipid metabolism, and sphingolipid metabolism were identified in the BC group and LS+BC group as being associated with the effect on lipid metabolism (Figures 7A,C). Other impacted pathways included beta-oxidation of very long-chain fatty acids, phosphatidylinositol phosphate metabolism, vitamin B6 metabolism, carnitine synthesis, and oxidation of branched chain fatty acids in BC group and LS+BC group (Figures 7D,F). Meanwhile, three main pathways were confirmed in the LS group compared to the HFD group, including sphingolipid metabolism, glycerophospholipid metabolism, and pentose and glucuronate interconversions (Figure 7B). The enrichment overview also highlighted the presence of malate-aspartate shuttle, gluconeogenesis, alpha linolenic acid, and linoleic acid metabolism and pentose phosphate pathway in LS group compared with the HFD group (Figure 7E).

**Table 2:** The result of pathway enrichment analysis of plasma in BC group *vs*. HFD group, LS group *vs*. HFD group and LS+BC group *vs*. HFD group*.

**Metabolic pathway**	**BC** ***vs***. **HFD**					**LS** ***vs***. **HFD**					**LS**+**BC** ***vs***. **HFD**				
	**Total**	**Hits**	**Raw p**	**-log(p)**	**Impact**	**Total**	**Hits**	**Raw p**	**-log(p)**	**Impact**	**Total**	**Hits**	**Raw p**	**-log(p)**	**Impact**
Glycerophospholipid metabolism	30	4	1.51E-04	8.80E+00	0.36	30	2	4.12E-02	3.19E+00	0.28	30	4	6.52E-04	7.34E+00	0.47
Sphingolipid metabolism	21	2	1.25E-02	4.38E+00	0.27	21	3	1.04E-02	4.57E+00	0.28	21	2	3.45E-02	3.37E+00	0.28
Linoleic acid metabolism	5	1	4.13E-02	3.19E+00	0.00	5	1	7.61E-02	2.58E+00	0.00	5	1	6.94E-02	2.67E+00	0.00
alpha-Linolenic acid metabolism	9	1	1.04E-01	2.26E+00	0.00	9	1	1.33E-01	2.02E+00	0.00	9	1	1.22E-01	2.11E+00	0.00
Glycosylphosphatidylinositol (GPI)- anchor biosynthesis	14	1	1.12E-01	2.19E+00	0.00	14	1	1.99E-01	1.61E+00	0.04	14	1	1.83E-01	1.70E+00	0.04
Arachidonic acid metabolism	36	1	2.64E-01	1.33E+00	0.00	36	1	4.38E-01	8.25E-01	0.00	36	1	4.08E-01	8.97E-01	0.00
Glycerolipid metabolism	16	1	1.27E-01	2.07E+00	0.01	-	-	-	-	-	16	1	2.29E-01	1.47E+00	0.02
Vitamin B6 metabolism	9	1	7.32E-02	2.61E+00	0.05	-	-	-	-	-	9	1	1.22E-01	2.11E+00	0.05
Phosphatidylinositol signaling system	28	1	2.12E-01	1.55E+00	0.00	-	-	-	-	-	-	-	-	-	-
Ascorbate and aldarate metabolism	-	-	-	-	-	9	1	7.61E-02	2.58E+00	0.00	-	-	-	-	-
Pentose and glucuronate interconversions	-	-	-	-	-	14	1	1.99E-01	1.61E+00	0.27	-	-	-	-	-
Terpenoid backbone biosynthesis	-	-	-	-	-	15	1	2.12E-01	1.55E+00	0.00	-	-	-	-	-
Starch and sucrose metabolism	-	-	-	-	-	23	1	3.07E-01	1.18E+00	0.04	-	-	-	-	-
Amino sugar and nucleotide sugar metabolism	-	-	-	-	-	37	1	4.47E-01	8.05E-01	0.00	-	-	-	-	-
Tryptophan metabolism	-	-	-	-	-	41	1	4.82E-01	7.30E-01	0.00	-	-	-	-	-
Drug metabolism - cytochrome P450	-	-	-	-	-	56	1	5.95E-01	5.19E-01	0.04	56	1	3.72E-01	9.89E-01	0.00
Biosynthesis of unsaturated fatty acids	-	-	-	-	-	42	1	4.90E-01	7.12E-01	0.00	42	1	4.58E-01	7.81E-01	0.00
Arginine and proline metabolism	-	-	-	-	-	-	-	-	-	-	44	1	4.74E-01	7.47E-01	0.01
Steroid hormone biosynthesis	-	-	-	-	-	-	-	-	-	-	70	1	6.44E-01	4.41E-01	0.00

*Total is the total number of compounds in the pathway; the Hits is the actually matched number from the user uploaded data; Raw p-value was calculated from the enrichment analysis; the Impact is the pathway impact value calculated from pathway topology analysis.

### The relationship of gut microbiota and serum lipid profiles in HFD-induced obese rats

**Figure 8:**
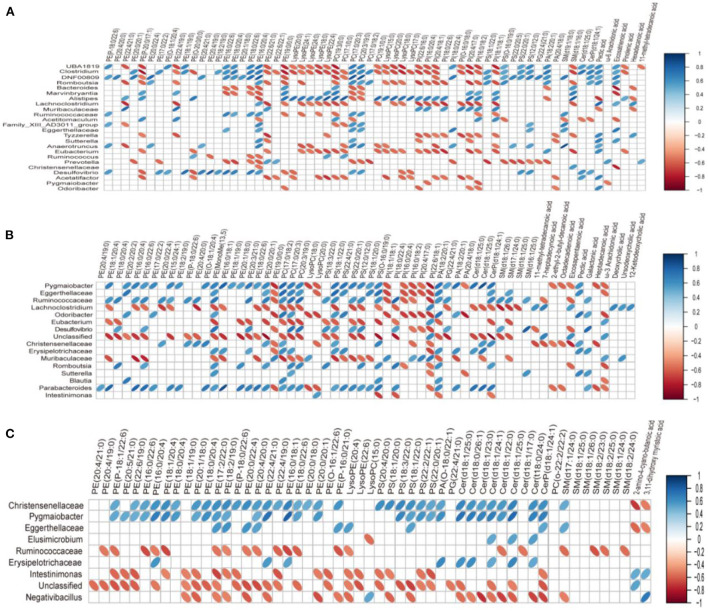
Correlation between lipid metabolism and gut microbiota in serum. **(A)** BC group *vs*. HFD group, **(B)** LS group *vs*. HFD group, **(C)** LS+BC group *vs*. HFD group. On the top of the graph is the name of metabolite, and on the left side is the name of association gut microbiota. Blue for positive correlation and red for negative correlation.

In order to study the co-variation of the gut microbiota and serum lipid profiles, heatmap visualization of the microbiota-lipid correlations is shown in Figure 8. The statistical correlations were detected between 24 genera of gut microbiota and 64 lipid metabolites between the BC group and HFD group (Figure 8A). The decreased PE and PC species including PE(16:0/20:4), PE(16:0/22:6), PE(18:0/20:4), PE(18:0/22:6), PC(17:0/20:3), and PC(20:3/19:0) were positively correlated with *UBA1819, Clostridium, DNF00809, Ruminococcaceae, Desulfovibrio* and negatively correlated with *Bacteroides, Muribaculaceae*, and *Prevotella*. Inversely, a positive correlation was detected between five LysoPE species [LysoPE(18:0), LysoPE(20:0), LysoPE(22:4), LysoPE(24:0) and LysoPE(24:1)], 4 LysoPC species [LysoPC(15:0), LysoPC(17:0), LysoPC(18:0) and LysoPC(20:0)], PC(19:3/0:0), PI(20:4/17:0) and *UBA1819, Clostridium, Lachnoclostridium, Eubacterium* in the BC group. Moreover, PE(16:0/18:1), PE(16:0/20:4), PE(16:0/22:6), PE(18:0/22:6), PE(18:1/20:4), PC(17:0/20:3), PC(20:3/19:0) and PS(12:0/12:0) were positively associated with *Pygmaiobacter, Ruminococcaceae, Parabacteroides* and negatively associated with *Lachnoclostridium* and *Muribaculaceae* in the LS+BC group (Figure 8C). In the LS group, only eight genera were detected to be associated with lipid metabolites. Among them, *Christensenellaceae* and *Pygmaiobacter* were positively correlated with most PE, PS, and Cer species, especially PE(16:0/18:1), PE(16:0/22:6), PE(18:0/22:6), PE(18:0/20:4), and PE(P-18:1/22:6), which were significantly decreased in the LS group compared to the HFD group (Figure 8B).

### Effect of BCPs treatment on glycerophospholipid metabolism and sphingolipid metabolism signaling pathway genes and protein expression in liver and adipose tissues of HFD-induced obese rats

**Figure 9:**
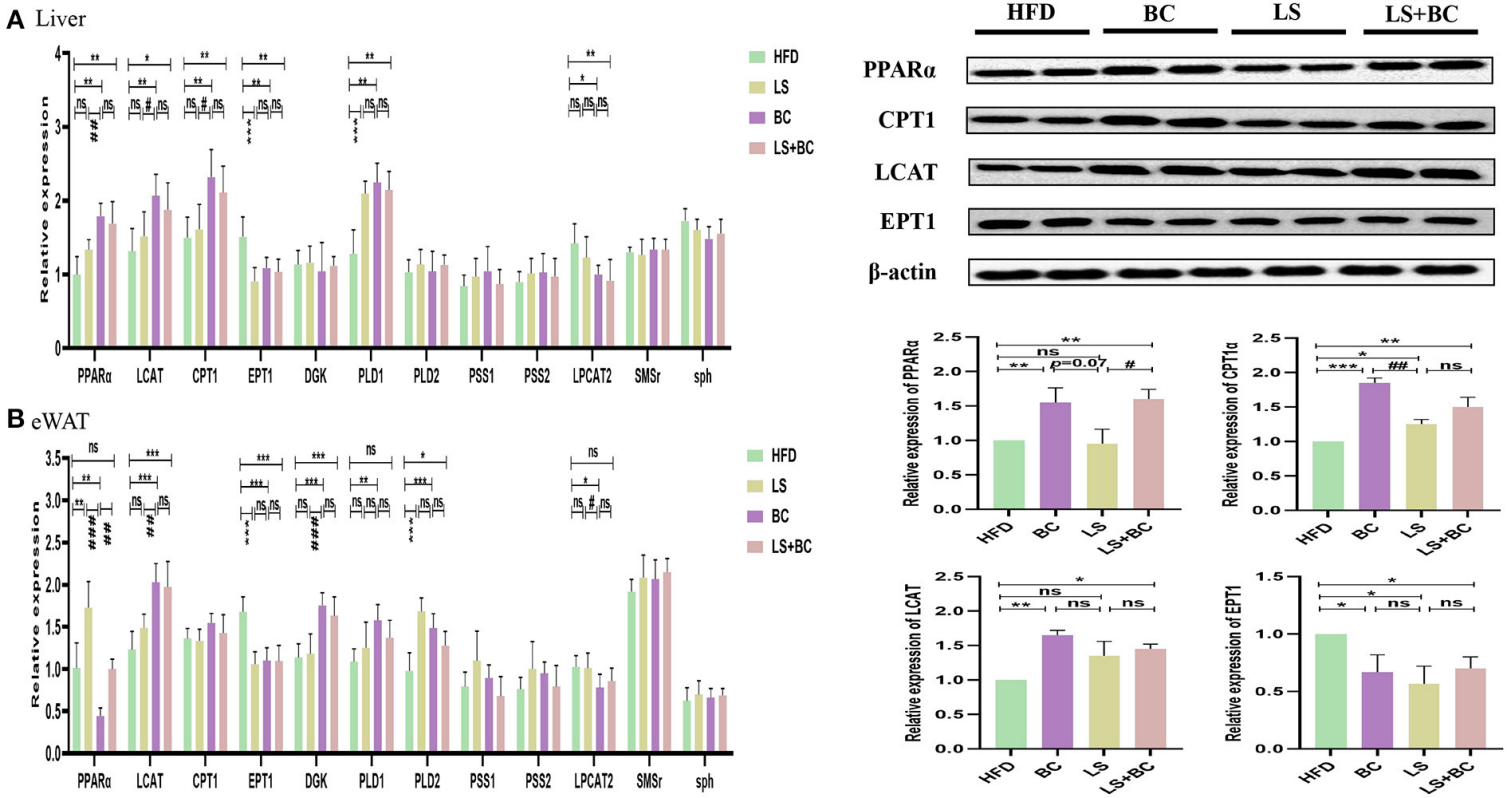
Effect of BCPs treatment on hepatic and eWAT gene expression linked to glycerophospholipid metabolism and sphingolipid metabolism in HFD-induced obese rats. The glycerophospholipid metabolism and sphingolipid metabolism pathway related genes were measured and β-actin gene was applied as a reference. **(A)** the mRNA expression in the liver; **(B)** the mRNA expression in the eWAT. The Values are means ± SEMs. *p < 0.05, **p < 0.01, and ***p < 0.001 compared with HFD group, ^#^*p* < 0.05, ^##^*p* < 0.01, ^###^*p* < 0.001 compared with BC group.

To determine the role of BCPs treatment in mediating glycerophospholipid metabolism and sphingolipid metabolism signaling pathway, we evaluated the relative expression levels of glycerophospholipid metabolism and sphingolipid metabolism-related genes. In the liver, the mRNA expression level of peroxisome proliferator-activated receptor alpha (PPARα), lecithin:cholesterol-acyl-transferase (LCAT), carnitine palmitoyltransferase 1 (CPT1), and phospholipase D1 (PLD1) were significantly up-regulated, whereas the mRNA expression of ethanolamine phosphotransferase 1 (EPT1) and lysophosphatidylcholine acyltransferase 2 (LPCAT2) was down-regulated in the BC group and LS+BC group compared to the HFD group (Figure 9A). There was no significant difference in the mRNA expression of diacylglycerol kinase (DGK), phospholipase D2 (PLD2), phosphatidylserine synthase 1 (PSS1), phosphatidylserine synthase 2 (PSS2), sphingomyelin synthase (SMS)-related protein (SMSr) and sphingomyelin phosphodiesterase (sph) among the four groups. In addition, after the simvastatin treatment, only PLD1 and EPT1 were significantly altered in HFD-induced obese rats. In eWAT, the mRNA expression of LCAT, DGK, and PLD2 were up-regulated, whereas the mRNA expression of EPT1 was down-regulated in the BC group and LS+BC group compared to the HFD group (Figure 9B). Moreover, BCPs supplementation increased the mRNA expression of PLD1 and reduced the mRNA expression of LPCAT2 and PPARα. After simvastatin treatment, the mRNA expression of EPT1 was lower and the mRNA expression of PPARα and PLD2 was increased in eWAT. Further, in the liver, the protein expression level of PPARα, CPT1, and LCAT was markedly increased and the protein expression level of EPT1 was decreased in the BC group and LS+BC group compared to the HFD group.

## Discussion

Obesity is associated with increasing the risk of many metabolic syndromes, including dyslipidemia, hepatic steatosis, inflammation, and other chronic disease symptoms. In this study, we investigated the potential effects of BCPs treatment on obesity and lipid metabolic disorders in HFD-induced obese rats. In particular, our results indicated that BCPs supplementation reduced overall body weight, liver, and white adipose tissue weight, weight gain, and alleviated dyslipidemia and hepatic steatosis in HFD-induced obese rats. Moreover, BCPs supplementation altered the composition and structure of the gut microbiota, which was significantly associated with lipid metabolism in HFD-induced obese rats. Furthermore, lipid classes and individual species were also identified through unbiased lipidomic analysis. Differential lipid metabolites were mainly enriched in glycerophospholipid metabolism and sphingolipid metabolism signaling pathway. Finally, we demonstrated that BCPs treatment significantly changed the mRNA and protein levels of PPARα, LCAT, CPT1, and EPT1 in the glycerophospholipid metabolism signaling pathway, which might be the underlying mechanisms involving BCPs treatment to alleviate obesity through reshaping gut microbiota and improving lipid metabolism disorders. Meanwhile, the results also indicated that both BCPs treatment alone and BCPs with low-dose simvastatin treatment have positive effects on obesity, which was better than low-dose simvastatin treatment alone.

Specifically, the body weight, liver weight, eWAT weight, iWAT weight, pWAT weight, and mWAT weight were observably decreased after BCPs treatment in HFD-induced obese rats (Figures 1A,B and Table 1). In the liver, BCPs treatment could reduce the TC and TG levels and reverse lipid accumulation. Meanwhile, the level of TC, TG, and LDL-C in serum was lower and the level of HDL-C was higher in the BC group compared to the HFD group (Table 1). Besides, simvastatin was applied as control and decreased the level of TC and LDL-C in serum by inhibiting the endogenous synthesis of cholesterol. Surprisingly, low-dose simvastatin treatment was better than high-dose simvastatin treatment in regard to reducing body weight and liver weight. Moreover, low-dose simvastatin treatment and high-dose simvastatin treatment failed to lower the level of TG and increase the level of HDL-C, which could not effectively alleviate obesity and hepatic steatosis in a dose-dependent manner. However, when BCPs were combined with low-dose simvastatin treatment, the effect of reducing body weight and serum TG level was superior to low-dose simvastatin treatment alone.

Lots of evidence has been gathered in support of the important role that the gut microbiota plays an important role in the pathogenesis of metabolic diseases such as obesity, insulin resistance, and type 2 diabetes (18). This study specifically evaluated the effect of BCPs treatment on gut microbiota in HFD-induced obese rats. There was no significant difference in the ACE index, Chao1 index, Shannon index, and Simpson index among these groups (Supplementary Figure 2). However, BCPs treatment observably reduced the F/B ratio in obese rats, which has been correlated with obesity and energy metabolic disorders (19). In particular, some reports have shown that several specific genera and/or species of gut microbiota could play a central role in the host's metabolism and obesity. The abundance of species *Bacteroides* and *Prevotella* was inversely associated with obesity and improved insulin sensitivity in mice (20). Furthermore, the fecal microbiota transplantation (FMT) from lean co-twins showed a higher abundance of the *Bacteroides* and *Prevotella*, as well as resulting in an increased level of short-chain fatty acids (2). *Akkermansia* had been identified to have a positive effect in reversing the metabolic disorders caused by a high-fat diet, including insulin resistance, diabetes, and adipose tissue inflammation (21). Henning et al. (22) have demonstrated that *Prevotella* and *Romboutsia* were significantly correlated with weight loss induced by tea extracts. However, the research results of Congying Chen et al. demonstrated that *Prevotella* activated host chronic inflammatory responses and increased host fat deposition significantly in pigs fed with commercial formula diets (23). In this study, the relative abundance of *Bacteroides, Prevotella, Romboutsia*, and *Akkermansia* were significantly decreased in the HFD group, whereas BCPs treatment could observably change these trends (Figure 3B). Besides, *Desulfovibrio* was one of the conditional pathogenic bacteria, which has been strongly correlated with hepatic inflammation and was enriched in samples of patients with T2DM (24). Compared with the HFD group, the relative abundance of *Desulfovibrio* and *Clostridium* in the BC group was significantly reduced (Figure 3B). The LEfSe results also demonstrated that BCPs treatment markedly improved the relative abundance of *Bacteroides* and *Prevotella* in HFD-induced obese rats (Figure 4A). Therefore, we concluded that alterations in the gut microbiota, especially the relative abundance of *Bacteroides, Prevotella, Romboutsia, and Akkermansia* play a crucial role in the overall beneficial effect of BCPs treatment in alleviating obesity, hepatic steatosis, and dyslipidemia. Additionally, the LEfSe results indicated that simvastatin treatment alone, whether low-dose or high-dose, could not significantly improve the structure of the gut microbiota in HFD-induced obese rats (Figure 4B and Supplementary Figure 3E). Furthermore, high-dose simvastatin treatment even reduced the relative abundance of some beneficial bacteria about inhibiting obesity (*Bacteroides* and *Akkermansia*) and increased the relative abundance of *Clostridium*, which have been positively correlated with obesity (25). We also found that BCPs combined with low-dose simvastatin treatment were better than low-dose simvastatin treatment alone for improving the structure of gut microbiota. The relative abundance of *Desulfovibrio, Ruminococcaceae_UCG-013*, and *Ruminococcaceae_NK4A214_group* was decreased, while the relative abundance of *Bacteroides, Prevotella*, and *Romboutsia* was increased in the LS+BC group compared to HFD group (Figure 3B). Shi et al. found that *Ruminococcaceae* was were enriched in the HFD-induced mice (26). Due to the results that BCPs combined with high-dose simvastatin treatment, as well as high-dose simvastatin treatment alone failed to alter the composition of beneficial bacteria in the intestine, we continued to study the anti-obesity effect in the HFD group, BC group, LS group, and LS+BC group. Through the PICRUSt analysis of gut microbiota, we found that the relative abundance of microbial genes involved in lipid metabolism pathways (CDP-diacylglycerol biosynthesis I pathway, CDP-diacylglycerol biosynthesis II pathway, phosphatidylglycerol biosynthesis I (plastidic) pathway, phosphatidylglycerol biosynthesis II (non-plastidic) pathway and super pathway of phospholipid biosynthesis I pathway) was markedly altered in the BC group and LS+BC group compared to the HFD group. Therefore, the next step was to study the effect of BCPs treatment on lipid metabolism in obese rats.

Lipid metabolism pathways are greatly complicated and associated with numerous diseases such as diabetes type 2, hyperlipidemia, obesity, insulin resistance, cardiovascular diseases, and so on (27–29). As traditional lipid risk factors, TC, TG, HDL-C, and LDL-C could not specifically explain the pathogenic mechanism of these diseases. Lipidomics is a new frontier of “omics” research, which provides much promise for a new generation of biomarkers to expand our understanding of the complexity of lipid dysregulation in obesity and metabolic diseases (30). Through serum lipidomics analysis, PE, PC, PS, SM, PI, Cer, LysoPE, LysoPC, and FAs and their conjugates were mainly altered in our study. Among them, the total PE, PC, PS, SM, and Cer species were decreased, whereas the total LysoPC, PI, FAs and conjugates, and secosteroids were elevated in the BC group and LS+BC group compared to the HFD group (Figures 5B,D). Pathway enrichment analysis results showed that differential lipid species were mainly enriched in glycerophospholipid metabolism and sphingolipid metabolism pathway (Figures 7A,C and Table 2). The results indicated that BCPs treatment and BCPs combined with simvastatin treatment may share a similar lipid metabolic signaling pathway (glycerophospholipid metabolism and sphingolipid metabolism) to alleviate the lipid metabolic disorders in HFD-induced obese rats. Furthermore, PC and PE are the most abundant phospholipids in all mammalian cell membranes and are implicated in many metabolic diseases (31). Several studies have found an increased PE level in high-fat diet-induced obese mice and monkeys (32, 33). However, some reports indicated that PE was negatively associated with many diseases, such as hepatic steatosis and diabetes (34). In our study, when the length of the acyl chain at the *sn*-1 position <20 carbons, the level of PE species was decreased in the BC group and LS+BC group. In detail, PE containing polyunsaturated fatty acyl (PUFA) chains at the *sn*-2 position, such as PE(16:0/20:4), PE(16:0/22:6), PE(18:0/20:4), and PE(18:0/22:6), were dramatically decreased in the BC group, LS group, and LS+BC group compared to the HFD group. However, PE (22:4/19:0), PE (22:4/21:0), PE (22:6/21:0), and PE (22:6/22:1) containing 22 carbons at the *sn*-1 position were elevated in the BC group compared to the HFD group (Figure 6A). The results demonstrated that the length and saturation of acyl chains in PE species influenced their biological functions. Similarly, PC species only containing one acyl chain, such as PC(17:1/0:0), PC(19:1/0:0), and PC(19:3/0:0) increased and reduced the risk of obesity in the BC group, whereas PC(17:0/18:2), PC(17:0/20:3), and PC(20:3/19:0) were decreased in the BC group and LS+BC group compared to the HFD group (Figure 6C). Our findings were consistent with previous studies which have demonstrated that specific PE or PC species rather than the total PE or PC levels influence obesity (28).

**Figure 10:**
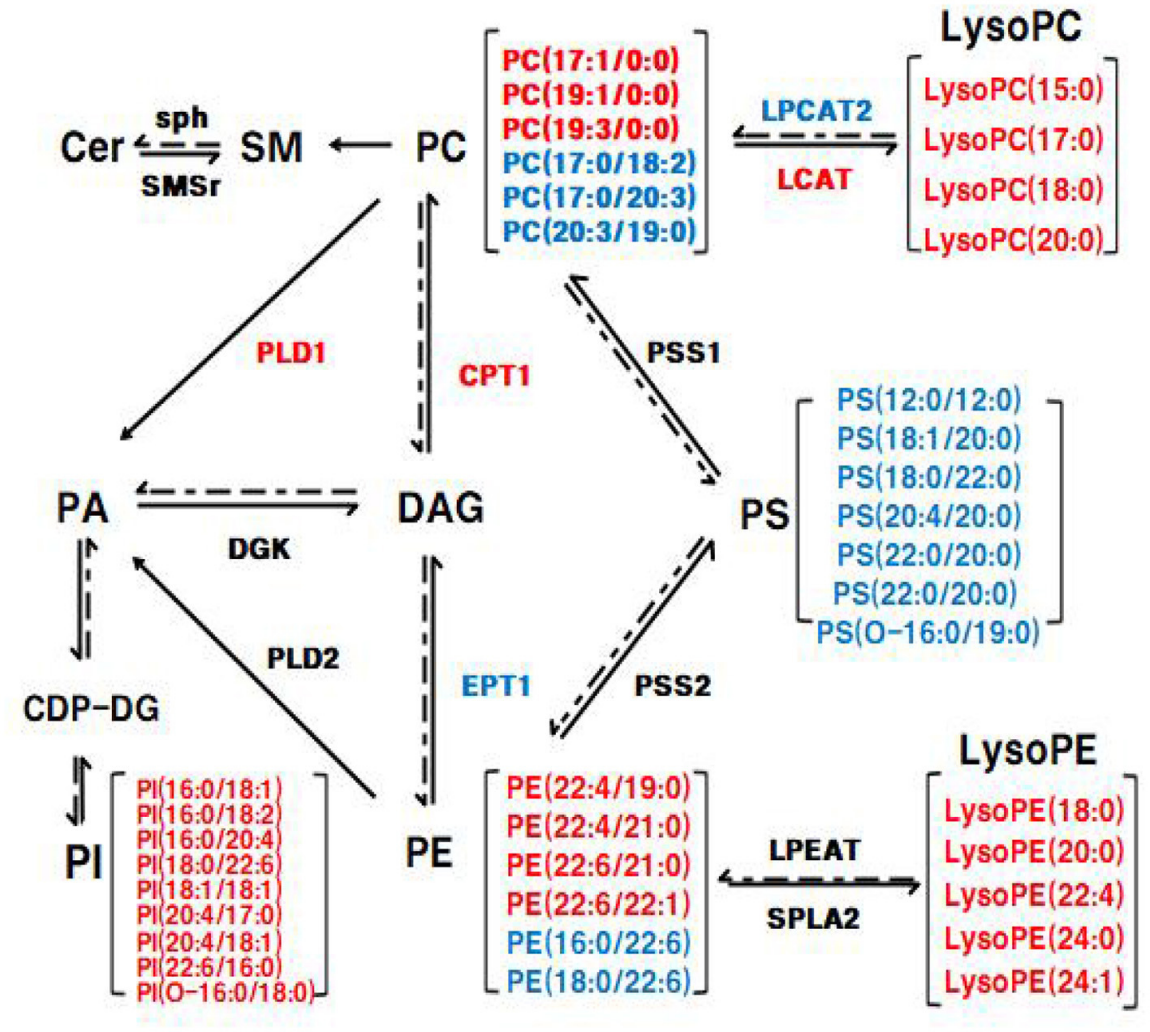
The Glycerophospholipid metabolism and sphingolipid metabolism in liver. The mRNA expression of genes in red was up-regulated and the mRNA expression of genes in blue was down-regulated in eWAT, respectively; the lipid level in red was up-regulated and the lipid level in blue was down-regulated in eWAT, respectively.

Moreover, as the important signaling molecules, the LysoPE and LysoPC are derived from PC and PE, respectively. Previous research has demonstrated that LysoPE and LysoPC (LysoPC(18:0), especially) may be negatively associated with risks of hypercholesterolemia and obesity (35, 36). In our study, five LysoPE species and four LysoPC species were increased in the BC group compared to the HFD group. Furthermore, LysoPE(24:0), LysoPE(24:1), LysoPC(18:0), and LysoPC(20:0) were observably increased in the BC group (FC>2) (Figure 6A). Compared to the HFD group, LysoPE species were not significantly altered, while 2 LysoPC species, LysoPC(18:0) and LysoPC(20:0), were significantly higher in the LS+BC group (Figure 6C). Besides, the levels of all LysoPC and LysoPE species were not changed between the LS group and HFD group (Figure 6B). In fact, previous studies have shown that LysoPC in total plasma was primarily contained in HDL particles (37). So we speculated that enrichment in LysoPC species [LysoPC(15:0), LysoPC(17:0), LysoPC(18:0), and LysoPC(20:0)] could result in the increased HDL-C level after BCPs treatment, thereby reducing the risk of obesity and dyslipidemia. In addition, Cer was primarily contained in LDL-C, and inhibition of the Cer biosynthesis pathways has been proven to confer therapeutic benefits in lots of metabolic diseases (38, 39). SM could be synthesized through PC and Cer species and the level of SM in plasma membrane directly influences cholesterol homeostasis (40). The reduction of SM inhibited cholesterol biosynthesis and alleviated LDL-C present in atherosclerotic plaques (41). In our study, the levels of seven SM species, nine Cer species, TC, and LDL-C in serum were simultaneously decreased in the LS group compared to the HFD group (Figure 6B). Previous studies have proven that statin could inhibit the HMG-CoA reductase activities and then decreased the TC and LDL-C levels (42). Here, we present new evidence for the correlation between simvastatin treatment and hypercholesteremia. Simvastatin treatment may decrease the levels of TC and LDL-C by regulating the SM and Cer metabolic pathways in HFD-induced obese rats. However, few SM and Cer species were altered in the BC group compared to the HFD group. In addition, the levels of PS species were decreased in the BC group, LS group, and LS+BC group compared to the HFD group. Some researchers have indicated that PS was synthesized by base exchange from PC or PE (Figure 10). In our study, most PC and PE species were reduced in the three groups, which may influence the PS synthesis. Interestingly, the levels of PI and PA species were increased in the BC group and LS+BC group compared to the HFD group. PA can be synthesized from 1,2-diacylglycerol (DAG) and converted to PI in two enzymatic steps (43). Shimizu et al. previously reported that body weight gain and the plasma levels of AST and cholesterol were suppressed after oral administration of PI suspension (44). However, serum-based PI exhibited a significant association with bipolar disorder risk and non-alcoholic fatty liver disease (45). Therefore, the effect of PI species on obesity and lipid metabolic disorders needs to be further investigated. Our results indicated that BCPs treatment has a great potential in the alleviation of lipid metabolic disorders in HFD-induced rats.

Furthermore, many studies have demonstrated the relationship between gut microbiota and the homeostasis of lipid metabolism in the host (3, 46). Here, our studies provided new evidence that the composition of gut microbiota was closely related to the lipid profiles after BCPs treatment. The increased relative abundance of *Clostridium, Ruminococcaceae_UCG-013*, and *Desulfovibrio* in HFD-induced obese rats were positively correlated with PE(16:0/20:4), PE(16:0/22:6), PE(18:0/20:4), PE(18:0/22:6), PC(17:0/20:3) and PC(20:3/19:0) in the BC group *vs*. HFD group (Figure 8A). However, *Bacteroides, Muribaculaceae*, and *Prevotella*, which have been reported to be reduced in obese individuals, were negatively correlated with PE(16:0/20:4), PE(16:0/22:6), PE(18:0/20:4), PE(18:0/22:6), PC(17:0/20:3) and PC(20:3/19:0) in BC group *vs*. HFD group. In addition, LysoPE(20:0), LysoPE(24:1), LysoPE(24:0), LysoPE(18:0), LysoPE(22:4), LysoPC(15:0), LysoPC(20:0), LysoPC(17:0), LysoPC(18:0), PC(19:3/0:0), PI(20:4/17:0) were negatively correlated with *UBA1819, Clostridium, Lachnoclostridium, Eubacterium* in the BC group *vs*. HFD group (Figure 8A). In the LS+BC group *vs*. HFD group, *Lachnoclostridium* and *Muribaculaceae*, which were beneficial to the inhibition of obesity, were negatively associated with PE(16:0/18:1), PE(16:0/20:4), PE(16:0/22:6), PE(18:0/22:6), PE(18:1/20:4), PC(17:0/20:3), PC(20:3/19:0), and PS(12:0/12:0). However, these lipids were positively associated with *Pygmaiobacter, Ruminococcaceae*, and *Parabacteroides* (Figure 8C).

To elucidate the underlying molecular mechanism of these effects, mRNA and protein expression levels of glycerophospholipid metabolism and sphingolipid metabolism signaling pathway related genes were measured in liver and adipose tissues. In the liver, the mRNA expression of PPARα and CPT1 were up-regulated in the BC group and LS+BC group compared to the HFD group (Figure 9A). PPARα has been shown to enhance free fatty acid β-oxidation and blunt the development of steatosis (47, 48). PPARα could be activated by natural and synthetic ligands such as PUFAs, eicosanoids, and so on (49). Through lipidomic analysis in serum, the long-chain unsaturated fatty acids (octadecadienoic acid, ω-6 arachidonic acid, eicosatrienoic acid and pinolenic acid) were significantly increased in the BC group and LS+BC group compared to the HFD group, which may induce the PPARα activation. CPT1, as a target gene of PPARα, is an essential enzyme in fatty acid ß-oxidation and is associated with hyperlipemia and obesity (48). Compared with the HFD group, the mRNA expression of PPARα and CPT1 was up-regulated in the BC group. We concluded that BCPs treatment could ameliorate hepatic steatosis *via* the PPARα/CPT1 pathway in HFD-induced obese rats.Moreover, the degradation of DAG occurs through the phosphorylation and cytidine diphosphate diacylglycerol (CDP-DAG) pathway by DGK and EPT1, generating PA and PE, respectively (50, 51). A previous study by Chibalin, A.V. et al. indicated that DAG content was increased and DGK activity was decreased, resulting in impaired insulin signaling and glucose metabolism in patients with type 2 diabetes (52). In our study, the mRNA expression of EPT1 was down-regulated, whereas the mRNA expression of DGK was not significantly altered in the BC group and LS+BC group compared to the HFD group. Besides, the levels of PC and LysoPC were regulated by LCAT and LPCAT2 genes through the “Lands Cycle” (Figure 10) (53). The up-regulated activity of LCAT in obese subjects potentially leads to an increase in circulating LysoPC levels. On the other hand, LPCAT2 is a key component of the Lands Cycle and catalyzes the esterification of LysoPC to form PC. After the BCPs treatment, the mRNA expression of LPCAT2 was inhibited and the mRNA expression of LCAT was increased in the liver, which indicated that more PC was converted to LysoPC. In addition, the available evidence has shown that PLD2 displays the same enzymatic activity as PLD1, catalyzing the hydrolysis of PC to produce free choline and PA. Jonathan Trujillo Viera et al. observed that PLD1^−/−^ and PLD2^−/−^ mice present elevated free fatty acids (FFAs) levels, which promote the development of overweight and diabetes (54). PSS1 and PSS2 catalyze the formation of PS from PE and PC, respectively (55). There was no significant difference in the mRNA expression of PLD1, PSS1, and PSS2 between the BC group and HFD group. Moreover, more DAG may be converted into PC rather than PE by upregulating the mRNA expression of CPT1 and downregulating the mRNA expression of EPT1, and then LysoPC was synthesized from PC through upregulation of the mRNA expression of LCAT and downregulation of the mRNA expression of LPCAT2 in the BC group and LS+BC group. As mentioned above, increased LysoPC content may be negatively associated with risks of hypercholesterolemia and obesity (35). In addition, activation of SMSr could increase levels of Cer and Cer were synthesized into SM by sph in the sphingolipid metabolism signaling pathway (56, 57). In our study, there was no significant difference in the mRNA expression of SMSr and sph among these groups. In eWAT, the mRNA expression of glycerophospholipid metabolism signaling pathway genes (LCAT, DGK, PLD1, PLD2) were up-regulated and EPT1 and LPCAT2 were down-regulated in the BC group compared to the HFD group. Furthermore, up-regulation mRNA expression of PLD1, PLD2, and DGK1 demonstrated that PA could be synthesized from PE, PC, and DAG in the BC group (Figure 9B). And then, PA is converted to PI at the endoplasmic reticulum for *De novo* PI synthesis (58). Therefore, in our study, three PA species and nine PI species were increased in the BC group compared to the HFD group (Figure 6A). Although the levels of PS species were decreased, the mRNA expression of PSS1 and PSS2 was not altered in the BC group and further investigation of this potential interaction is needed. Besides, we found that simvastatin treatment failed to change the mRNA expression of glycerophospholipid metabolism signaling pathway genes in the liver. In addition, the effect of BCPs combined with the simvastatin treatment was similar to that of BCPs treatment alone in HFD-induced obese rats, which could improve fat accumulation *via* the PPARα/CPT1 pathway in the liver. Meanwhile, BCPs combined with simvastatin treatment accelerated lipid transformation through up-regulation of the mRNA expression of LCAT, DGK, and PLD2 and down-regulation of the mRNA expression of EPT1 and LPCAT2 in eWAT. Regrettably, we did not identify the characteristic compounds and elucidate the bioconversion pathway after BCPs treatment in HFD-induced rats. It would further explain the anti-obesity mechanism of BCPs and their metabolites in HFD-induced rats.

## Conclusion

In summary, these findings revealed that BCPs supplementation significantly alleviated obesity and lipid metabolic dysfunctions in a rats model of HFD-induced obesity. Meanwhile, BCPs supplementation in HFD-induced obese rats increased the relative abundance of anti-obesity bacteria such as genera *Bacteroides, Prevotella, Romboutsia*, and *Akkermansia* and reduced the relative abundance of genera *Desulfovibrio* and *Clostridium*. Moreover, lipidomics analysis indicated that the total number of PE, PC, PS, SM, and Cer species decreased, whereas the total LysoPC, PI, FAs and conjugates, and secosteroids were elevated in the BC group compared to the HFD group. These lipid species, especially PE(16:0/22:6), PE(18:0/22:6), PC(20:3/19:0), LysoPE(24:0), LysoPE(24:1), and LysoPC(20:0), may be candidates for the diagnosis and study of obesity-related diseases. Furthermore, these dominant bacterial taxa altered by BCPs treatment were significantly associated with the lipid metabolic species in HFD-induced obese rats. In addition, the results indicated that BCPs treatment could ameliorate the disorder of lipid metabolism by regulating the mRNA and protein expression of genes related to the glycerophospholipid metabolism pathway in HFD-induced obese rats. The mRNA and protein expression of PPARα, CPT1, EPT1, and LCAT was significantly altered after BCPs treatment. Meanwhile, the effect of BCPs combined with simvastatin treatment on suppressing obesity and improving lipid metabolic disorders was similar to that of BCPs treatment, which was better than simvastatin treatment alone. Our findings provide evidence supporting the beneficial effects of BCPs intake on ameliorating functional disorders related to obesity and lipid metabolism.

## Data availability statement

The data presented in the study are deposited in the NCBI Sequence Read Archive (SRA) repository, accession number PRJNA841446.

## Ethics statement

The animal study experiments adhered to the China Institutional Animal Care Use Committee and were licensed by the Ethics Committee of Beijing Laboratory Animal Research Center (Qualified number: BLARC-2018-A033).

## Author contributions

A-dS and YZ conceived and designed the experiments. YZ performed the experiments. Y-lW, P-jC, IK, X-lQ, L-jZ, S-yZ, and YZ analyzed the data. Y-hX, C-lJ, YZ, XH, and A-dS wrote the paper. All authors read and approved the final manuscript.

## Funding

This present investigation was supported by the National Natural Science Foundation of China (31871817 and 32172222) and National Key R&D Plan (2016YFD0400302).

## Conflict of interest

The authors declare that the research was conducted in the absence of any commercial or financial relationships that could be construed as a potential conflict of interest.

## Publisher's note

All claims expressed in this article are solely those of the authors and do not necessarily represent those of their affiliated organizations, or those of the publisher, the editors and the reviewers. Any product that may be evaluated in this article, or claim that may be made by its manufacturer, is not guaranteed or endorsed by the publisher.
