# Supplementary material for: Modulation of the gut microbiota and lipidomic profiles by black chokeberry (Aronia melanocarpa L.) polyphenols via the glycerophospholipid metabolism signaling pathway
Source: Front Nutr. 2022 Aug 4;9:913729. doi: 10.3389/fnut.2022.913729 (PMC9387202; doi:10.3389/fnut.2022.913729)
Supplement: Supplementary file 1 [file Data_Sheet_1.PDF]

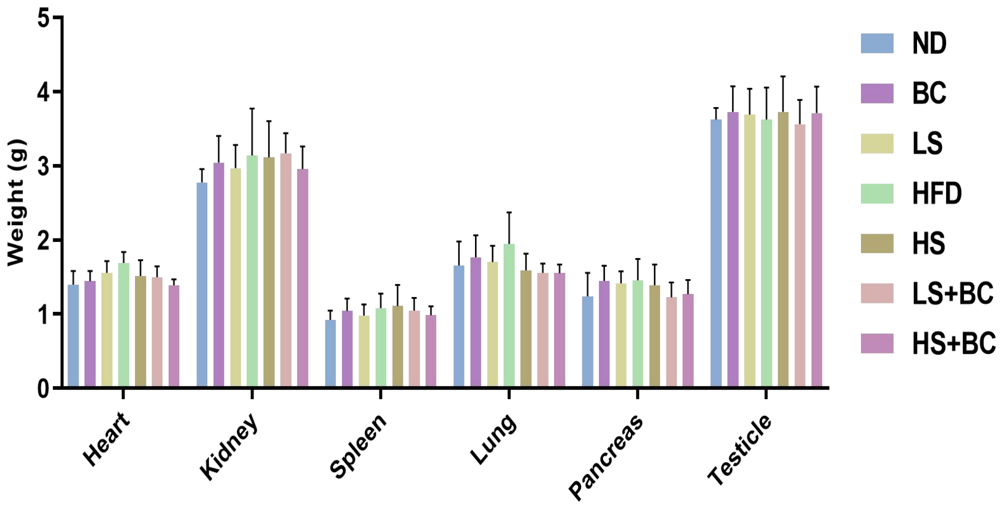

Supplementary Fig.1 The weight (g) of heart, kidney, spleen, lung, pancreas and testicle in the ND group, HFD group, BC group, LS group, HS group, LS+BC group and HS+BC group. Values are means ± SEMs.

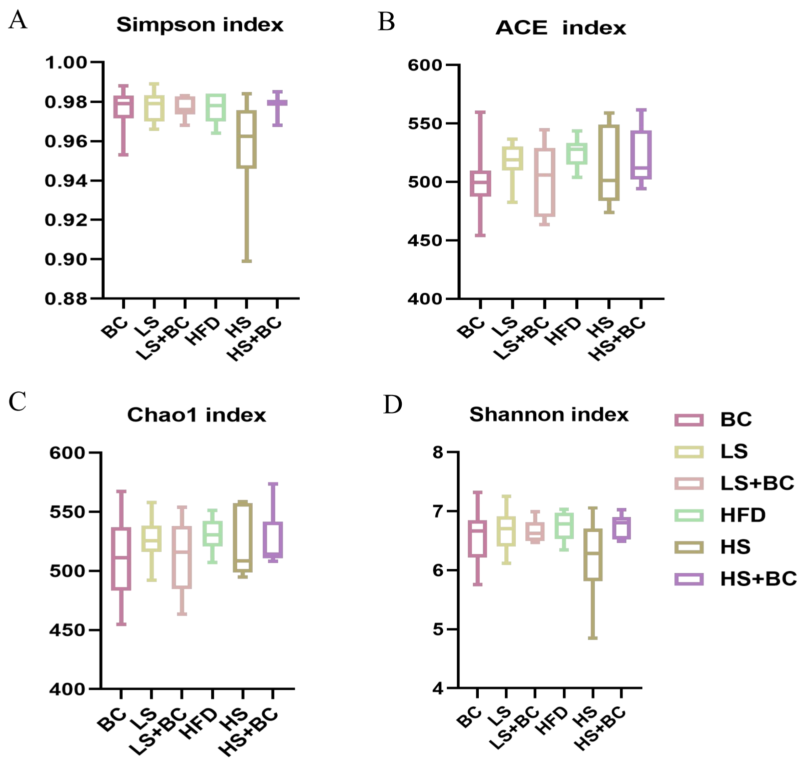

Supplementary Fig.2 (A) The ACE index, (B) Chao1 index, (C) Shannon index and (D) Simpson index in the BC group, LS group, HFD group, HS group, LS+BC group and HS+BC group after diet intervention.

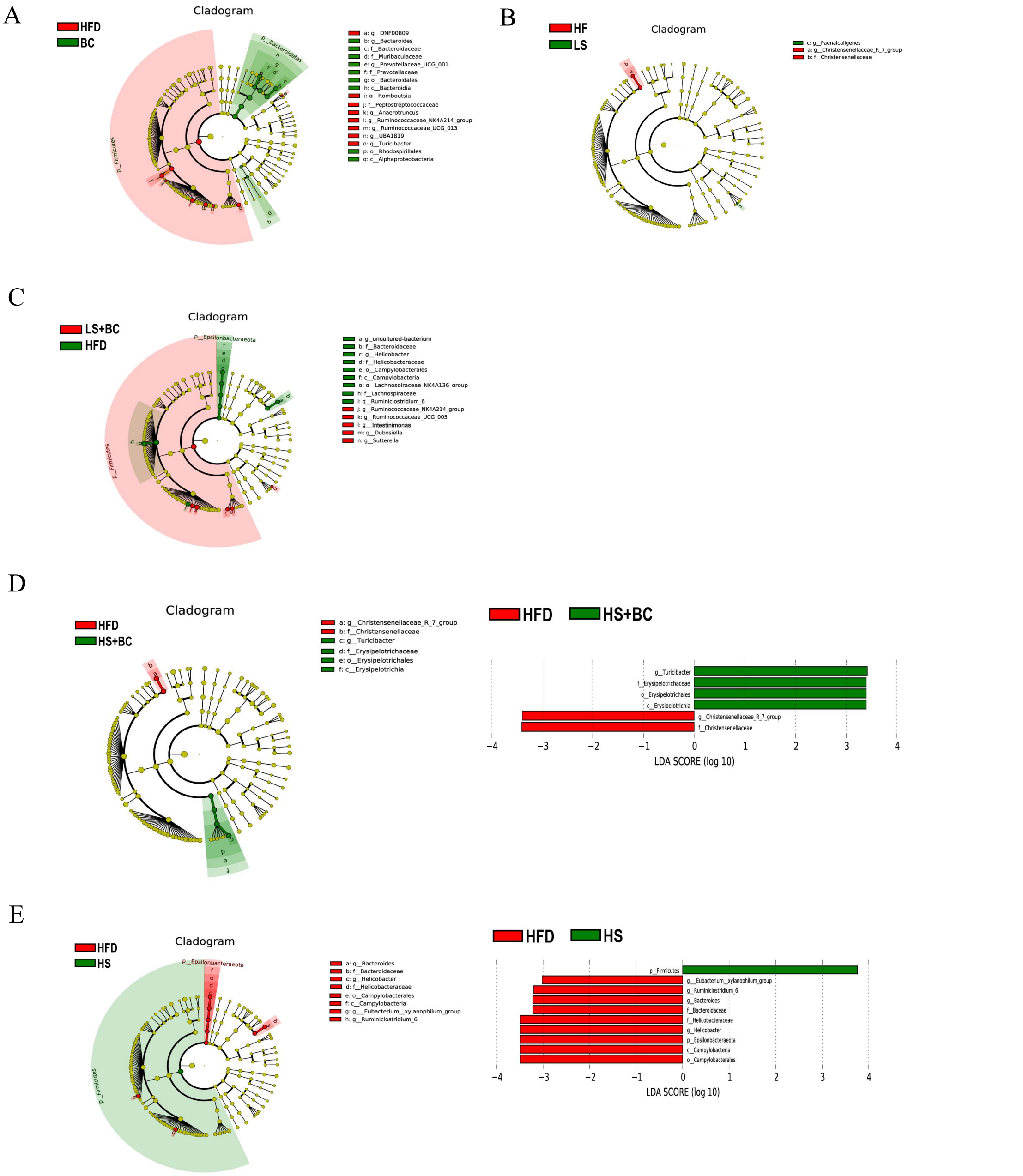

Supplementary Fig.3 LEfSe analyses of gut microbiota between the two groups: (A) BC *vs.* HFD, (B) LS *vs.* HFD, (C) LS+BC *vs.* HFD, (D) HS+BC *vs.* HFD, (E) HS *vs.* HFD.

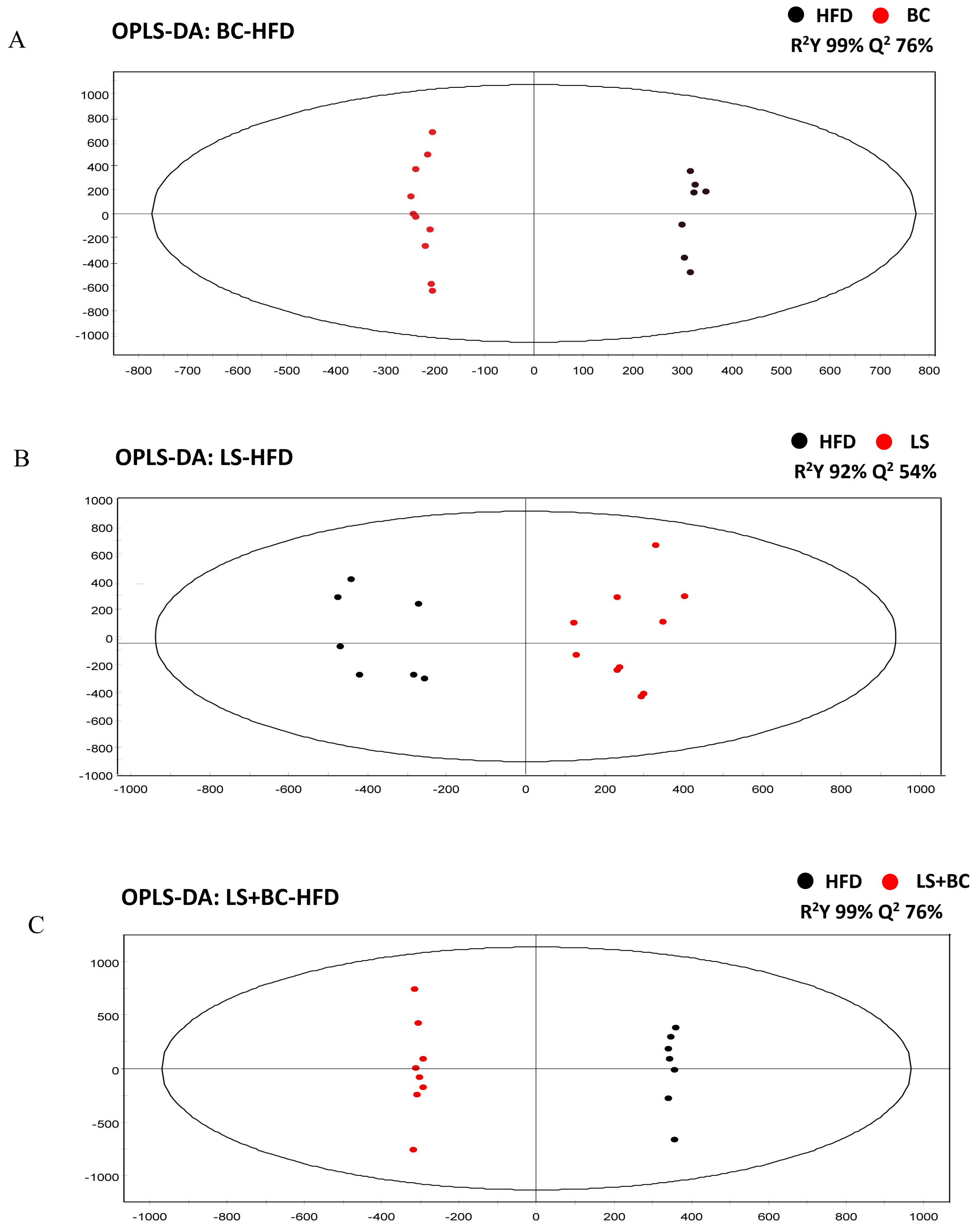

Supplementary Fig.4 Orthogonal projections to latent structures discriminant analysis (OPLS-DA) score plots of lipid profiles in serum. Colour gradient and circle size indicate the significance of the pathway ranked by p-value (yellow: higher p-values and red: lower p-values) and pathway impact score (the larger the circle the higher the impact score), respectively. (A): BC group *vs.* HFD group, (B): LS group *vs.* HFD group, (C): LS+BC group *vs.* HFD group.
